# Deciphering the Tubulin Language: Molecular Determinants and Readout Mechanisms of the Tubulin Code in Neurons

**DOI:** 10.3390/ijms24032781

**Published:** 2023-02-01

**Authors:** Riccardo Zocchi, Claudia Compagnucci, Enrico Bertini, Antonella Sferra

**Affiliations:** 1 Unit of Neuromuscular Disorders, Translational Pediatrics and Clinical Genetics, Bambino Gesù Children’s Hospital, IRCCS, 00146 Rome, Italy; 2 Molecular Genetics and Functional Genomics, Bambino Gesù Children’s Research Hospital, IRCCS, 00146 Rome, Italy

**Keywords:** microtubules, tubulins, tubulin post-translational modifications, tubulin code, neurons, axonogenesis, dendritogenesis, neuronal polarity, neuronal migration, microtubule associated proteins, motor proteins

## Abstract

Microtubules (MTs) are dynamic components of the cell cytoskeleton involved in several cellular functions, such as structural support, migration and intracellular trafficking. Despite their high similarity, MTs have functional heterogeneity that is generated by the incorporation into the MT lattice of different tubulin gene products and by their post-translational modifications (PTMs). Such regulations, besides modulating the tubulin composition of MTs, create on their surface a “biochemical code” that is translated, through the action of protein effectors, into specific MT-based functions. This code, known as “tubulin code”, plays an important role in neuronal cells, whose highly specialized morphologies and activities depend on the correct functioning of the MT cytoskeleton and on its interplay with a myriad of MT-interacting proteins. In recent years, a growing number of mutations in genes encoding for tubulins, MT-interacting proteins and enzymes that post-translationally modify MTs, which are the main players of the tubulin code, have been linked to neurodegenerative processes or abnormalities in neural migration, differentiation and connectivity. Nevertheless, the exact molecular mechanisms through which the cell writes and, downstream, MT-interacting proteins decipher the tubulin code are still largely uncharted. The purpose of this review is to describe the molecular determinants and the readout mechanisms of the tubulin code, and briefly elucidate how they coordinate MT behavior during critical neuronal events, such as neuron migration, maturation and axonal transport.

## 1. Introduction

Microtubules (MTs) are key components of the cell cytoskeleton, the dynamic network of interlinking filamentous proteins occurring in the cytoplasm of all cell types [1]. They are non covalent polymers composed of heterodimers of α- and β-tubulin that align head to tail to form a protofilament in which an α-tubulin subunit is exposed at one end, and a β-tubulin subunit at the other [2]. Protofilaments are then assembled laterally, in the same orientation, to form a hollow cylindrical structure that constitute the MT [3,4]. The directional alignment of the α- and β-tubulins into the protofilaments make MTs intrinsically polarized structures, in which a fast-growing plus-end (β-tubulin) and a slow-growing minus-end (α-tubulin) can be distinguished [5].

Within the cell, MTs do not reach a steady-state length but alternate periods of rapid polymerization with periods of shrinkage, in a process known as “dynamic instability”, that allows MTs to be swiftly remodeled in response to cellular needs.

In almost all cells, the α-tubulin minus-end is anchored to nucleating factors (such as the γ-tubulin ring complex) that act as template for the α- and β-tubulin heterodimers to start MT polymerization, so most of these dynamics occur mainly at the MT plus-end [6].

Despite their high similarity, MTs have a great functional heterogeneity, which is generated by the expression of different tubulin isotypes, which are cellular and temporally regulated, and by their PTMs [7]. Together, these regulatory mechanisms generate on both outer and inner surfaces of MTs a set of combinatorial information, known as the “tubulin code”, whose function is to adapt MTs to specialized cellular functions [8,9]. MT complexity increases significantly if we consider that the combination of tubulins and PTMs generates, within the cell, a large number of MT sub-populations with different structural, physical and dynamic parameters as well as protein affinity.

More than any other cell type, neurons rely on MT cytoskeleton to perform their functions. The creation and the proper functioning of complex neuronal structures, such as growth cones, axon and dendrites, depend on the reorganization of MTs [10]. Moreover, the correct coordination of MTs allows newly formed neurons to migrate appropriately towards specific brain regions [11] and provides for the maintenance of neuronal connectivity, supporting neuronal synapses through the long-distance delivery of vesicles, protein and organelles [12].

Nevertheless, how the tubulin code generates the information necessary to coordinate MT cytoskeleton during these events is, for some ways, still elusive.

## 2. Molecular Components of the Tubulin Code

MTs can be considered “mosaic structures” that are assembled from mixtures of different α- and β-tubulin isotypes. In humans, at least eight α-tubulin and nine β-tubulin isotypes have been identified [13], showing a remarkable degree of homology, with more than 90–95% of amino acid similarity for α-tubulins and 80–85% for β-tubulins [14].

All tubulin isotypes present a tripartite structure with: (i) a N-terminal nucleotide-binding domain, involved in the GTP binding; (ii) a middle region involved in longitudinal and lateral dimers interaction; (iii) an acid C-terminal domain, responsible for the binding of MT-interacting proteins and also the site of the major PTMs [15]. Most of the amino acid sequence differences between tubulin isotypes occurs in the C-terminal region; these changes provide the first structural basis for the generation of different MT population [14].

Based on their general degree of similarity, it was initially assumed that tubulins exert redundant functions. Currently, it is well established that different tubulin isotypes can assemble into discrete MT species with different functional significances, a concept known as the “multi tubulin hypothesis” [16]. The incorporation of different isotypes can affect the architecture of the α- and β-tubulin heterodimer, changing the positioning of elements that impact on MT functional properties [8,17] or modify structural features of MTs, such as their protofilament number. Tubulin heterodimers composed of α1B-tubulin and β2B-tubulin, for example, preferentially assemble in vitro into 14-protofilament MTs, while those constituted by α1B-tubulin and β3-tubulin mostly assemble into 13-protofilament MTs [18]. Furthermore, recent studies have shown that tubulin isotypes present different dynamic parameters in vitro [18,19,20,21], and changes in tubulin combination (ratio and concentration) can affect MT mechanics in vivo [17,18].

Tubulin isotypes are differentially expressed in neurons. 

Among β-tubulin isotypes, TUBB3 has been considered a marker of neuronal MTs and neurons in general. It is constitutively expressed in all neuronal cells [22] and involved in the processes of axon guidance and regeneration [22,23]. Its levels raise during neurogenesis and correlates with an increased MT turnover; MTs enriched in this tubulin, in fact, undergo catastrophe more frequently than that composed of other tubulin isotypes [24]. 

In addition to TUBB3, at least eight tubulins (TUBA1A, TUBB2A, TUBB2B, TUBB, TUBA4A, TUBB4A, and TUBG1) have been reported to be expressed in neurons [25,26,27,28,29,30,31,32,33]; mutations in genes encoding for neuronal tubulin isotypes give rise to a wide class of neurodevelopmental and neurodegenerative disorders known as “tubulinopathies” [34]. The heterogeneity of the neurological phenotypes associated with this class of diseases highlights the specific function and the spatio-temporal expression of each tubulin during brain development [35]. 

Unfortunately, our knowledge about the expression pattern of tubulins during brain maturation is still fragmentary, due to their high homology that leads to tubulin antibody promiscuity and makes difficult to track tubulin single protein expression. Very recently, in the attempt to explain how tubulin isotypes are regulated during brain development, Hausrat and colleagues have quantified both gene and protein levels of endogenous α- and β-tubulins in different regions of mouse brain, during its pre- and post-natal development [35]. They demonstrated that among α-tubulin isotypes, *Tuba1a*, *Tuba1b*, and *Tuba1c* have a prominent expression in the hippocampus at embryonic stage, while *Tuba4a* is the only α-tubulin that significantly increases during brain post-natal development. Accordingly, with its post-natal expression, mutations in the *TUBA4A* human gene are responsible for the amyotrophic lateral sclerosis, a late-onset disease characterized by adult-onset upper and lower motor neuron degeneration [36].

Among β-tubulins, instead, *Tubb2a* was reported to be the major tubulin expressed at later embryonic stages and around birth of mouse. Moreover, Hausrat and colleagues also showed that all β-tubulin isotypes, with the exception of *Tubb4a*, were significantly reduced in post-natal brains [35]. Interestingly, also mutations in *TUBB4A* cause a late adult-onset phenotype in humans, known as torsion dystonia type 4, characterized by progressive generalized dystonia [37].

Likewise, our knowledge about the local expression of tubulins in neurons is still elusive. Recently, the RNA of *Tubb2b* was found in association with the MT plus-end tracking protein (+TIP) Adenomatous polyposis coli at the growth cone periphery of cultured dorsal root ganglion (DRG) neurons, suggesting a role of this tubulin isotype in this neuronal compartment [38].

Tubulin isotypes are subjected to multiple enzymatic PTMs that diversify both the outer and the luminal surface of MTs [39]. Within the cell, these modifications label distinct MT populations, giving rise to additional levels of information. Several tubulin PTMs have been identified so far. Many of them are highly conserved throughout eukaryotic species, highlighting the functional relevance of these modifications (Table 1).

Numerous experimental efforts in recent years have made possible to understand, at least in part, the biological significance of many of these modifications.

In this section, we will briefly describe the tyrosination/detyrosination cycle of α-tubulin, the acetylation of K40 on α-tubulin, and the polyglutamylation and the polyamination of α- and β-tubulin.

Tubulin detyrosination was the first PTM to be discovered [89]. It consists of the enzymatic removal of the Y from the C-terminus of α-tubulin and is catalyzed by the recently discovered tubulin detyrosinases VASH1 1 and 2 [53,90]. This is a reversible reaction and the addition of the removed Y to the soluble tubulin is catalyzed by TTL [52,91,92]. 

MTs enriched in detyrosinated α-tubulin are more resistant to nocodazole in living cells, and to depolymerization by dilution in detergent-permeabilized cell models [51]

Detyrosinated tubulin can be further converted by CCP4, CCP5 and CCP6 into Δ2-tubulin, through the removal of the penultimate E in the C-terminal domain of the α-tubulin [55,69]. This is an irreversible PTM restricted to very stable MTs and is considered the final stage of α-tubulin maturation in long-lived polymers [69]

**Figure 1:**
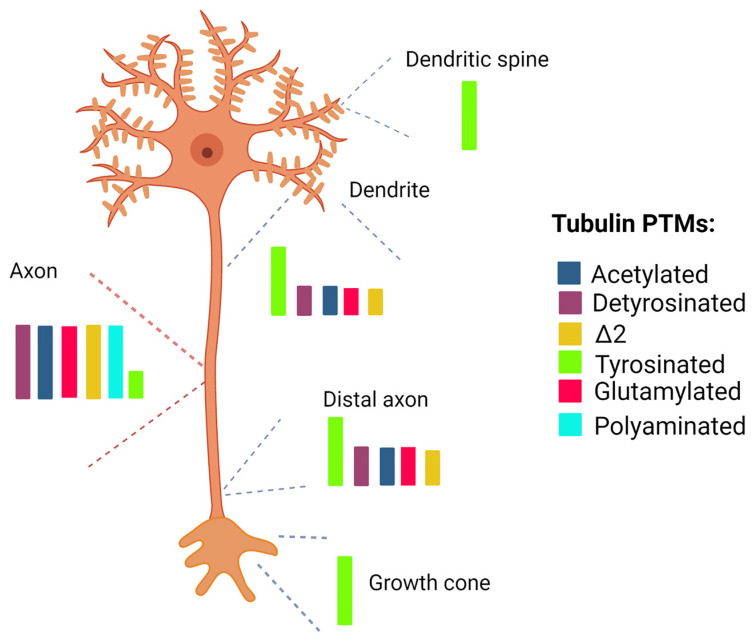
Tubulin PTMs in different neuronal compartments. Tubulin acetylation, glutamylation, polyamination and detyrosination are elevated in the axon of neuron. These modifications are low in the distal axon and absent in the growth cone. Low levels of tyrosinated tubulin are detected in the axon. Tubulin tyrosination is mainly detected in the distal region of the axon and in the growth cone, consistent with the presence of highly dynamic MTs in these compartments. High level of tyrosinated tubulins and low level of glutamylated, acetylated, tyrosinated, detyrosinated and Δ2-tubulins are detected in dendrites. Labile and mainly tyrosinated populations of MTs are localized in dendritic spines. The figure has been created in “BioRender.com”.

Detyrosinated and Δ2-tubulin are prominent modifications in neurons and found on all MTs except those of the growth cone [93] (Figure 1).

Moreover, detyrosinated tubulins are enriched in axonal MTs of developing neurons [94] and positively correlates with the extent of axon outgrowth, since its depletion increases axon outgrowth in cultured hippocampal neurons [95].

Differently, tubulin tyrosination is considered a marker of labile MTs [96]. In vivo studies have demonstrated that tyrosinated MTs undergo turnover within minutes [97]. Moreover, in vitro experiments have shown that in cultured MEF cells, tyrosinated MTs are preferentially bound by the kinesin Kif2C, a motor-like protein that induces the depolymerization of MT ends, demonstrating that MTs enriched in this PTM are more subjected to disassembly [98].

**Figure 2:**
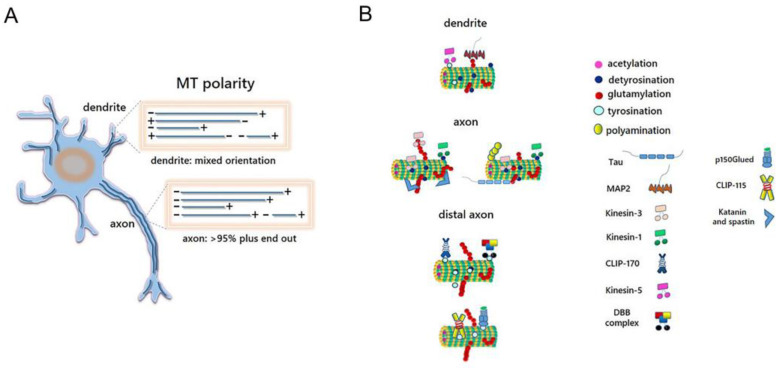
MT polarity and MT-interacting protein in different neuronal compartment. (**A**) In axon, MTs have uniform polarity, and more than 95% of MTs are aligned with their plus-ends pointing toward the tip of the axon. In dendrites instead, MTs have polarity. (**B**) In different neuronal compartment, the binding of MT-interacting proteins occurs on a subset of MTs labeled with specific PTMs. The plus-end-directed cargo transport of Kinesin-1 and Kinesin-3 occurs on acetylated and detyrosinated MTs in the axon. In this neuronal compartment, Kinesin-3 also interacts with polyglutamylated chains on the C-terminal domain of α-and β-tubulins. The severing enzymes katanin and spastin bind polyglutamylated MTs. Tau instead preferentially binds tubulins with moderate levels of glutamylation. In the distal axon, the DDB complex and the TIPs+ CLIP-170, CLIP-115 and p150 Glued selectively bind tyrosinated MTs. In dendrites, Kinesin-5 binds tyrosinated MTs while MAP2 interacts with MTs with low level of glutamylation.

Tyrosinated MTs are particularly abundant in the distal end of the axon, including the growth cone [99,100] (Figure 1) where they are necessary for the recruitment of +TIPs [101] (Figure 2). Interestingly, the initiation zone of the retrograde transport in the distal axon coincides with a region enriched in tyrosinated tubulin [102,103]. Recently, in vitro live-cell assays have demonstrated that tubulin tyrosination is implicated in the spatio-temporal regulation of the retrograde transport initiation in neurons [101].

The balance between tyrosinated and detyrosinated tubulin is tightly regulated during the early neuronal development, and the excessive detyrosination of neuronal MTs, caused by the inactivation of the gene encoding for the TTL enzyme, has been shown to cause neurodevelopmental defects and perinatal death in mice [104].

Analogously to detyrosination, the acetylation of K40 on α-tubulin have been reported to accumulate on long-lived MTs [41]. The peculiarity of this modification is that it marks the luminal surface of MTs [105]. The enzymatic addition of the acetyl moiety to K40 is catalyzed by the ATAT1 enzyme [106], while the reverse reaction is carried out by the deacetylases HDAC6 and SIRT2 [42,43]. In vitro reconstitution experiments have shown that, in addition to prevent MT depolymerization, α-tubulin acetylation protects MTs from mechanical breakage [45]. Indeed, the addition of the acetyl group on the luminal K40 changes the structural conformation of the flexible loop containing this residue, improving MT resilience and mechanical properties and, thus, stabilizing MTs [44,45,46].

Tubulin acetylation restricts axon overbranching and overgrowth by dampening MT dynamics in mice cerebral cortex and in cultured hippocampal neurons [107]; moreover, the loss of α-tubulin acetylation in cultured neurons promotes MT debundling, their invasion into filopodia and growth cones, and the plus-end dynamics along axons [107]. 

Tubulin polyglutamylation, consisting of the addition of E side chains of different length on the C-terminal domain of both α- and β-tubulins, predominantly impacts on MAP association [62]. Polyglutamylation is the most abundant modification in the nervous system, and is catalyzed by nine TTL enzymes [60]. The reverse reaction of E removal is instead catalyzed by all members of CCPs [55,61].

The addition of long side chains of E stimulates MT disassembly both in vitro and in vivo [108] and enhances, in vitro, the activity of the MT-severing enzyme spastin and katanin [108,109]. Thus, it was proposed that this PTM may modulate both MT mass and dynamics within the cell [110].

Axonal MTs are enriched in long-chain glutamylated tubulins while short-chain glutamylation occurs preferentially in dendritic MTs [111] (Figure 2). Moreover, α- and β-tubulins have been reported to be differentially glutamylated during the different stages of neuronal development; the levels of β-tubulin glutamylation indeed increase during in vitro neurogenesis whereas glutamylated α-tubulin is more abundant in cultured young neurons [60,112].

Imbalance in the level of tubulin glutamylation due to the inactivation of the *CCP1* gene, encoding for the CCP1 enzyme that removes E residues from polyglutamyl chains of tubulin, causes in *pcd* mice a neurodegenerative phenotype characterized by cerebellar ataxia and locomotor deficit [113]. In this animal model, the loss of CCP1 results in high levels of tubulin glutamylation in Purkinje cells that causes their post-natal degeneration [55] Recently, mutations in the *CCP1* human gene, that lead to the absence of a functional CCP1 protein, have been described also in patients affected by infantile-onset neurodegeneration involving the cerebellum, spinal motor neurons, and peripheral nerves [114].

Lastly, tubulin polyamination, consisting of the addition of amines to Q residues of both α- and β-tubulins, has been recently identified as a PTM enriched in axonal MTs (Figure 1) and critical for their stabilization [68].

It is an irreversible PTM catalyzed by TG1, TG2, TG3 and TG6 enzymes [115,116] and primarily occurs on amino acid residues at polymerization interfaces. The highly conserved Q15 on β-tubulin, located near the GTP-binding domain, is the main residue to be polyaminated [68,69]. Additional polyamination sites have been identified on α-tubulin (Table 1); these residues are located in the proximity of the GTP pocket, at the interface between neighboring protofilaments or with γ-tubulin at the nucleation site [68]. Considering the localization of these amino acid sites, tubulin polyamination has been predicted to have a role in MT lattice stabilization or nucleation [68,69]. 

Polyaminated MTs are highly stable to ice cold or calcium induced depolymerization condition; moreover, the inhibition of polyamine synthesis or transglutaminase activity significantly decreases MT stability both in vitro and in vivo [68]. The inhibition of ornithine decarboxylase, the main enzyme involved in polyamine biosynthesis in rats, reduces the formation of cold-insoluble tubulin in the axons of the rat optic nerve. Furthermore, the treatment of cultured SY5Y neuroblastoma cells with the irreversible transglutaminase inhibitor IR072 significantly decreases neurite outgrowth. Moreover, in vitro experiments demonstrated that the presence of polyamines reduces the solubility of tubulin and MTs as the bulk of modified tubulins were pelleted, according to an increase in their stability [68].

## 3. Readout Mechanisms of the Tubulin Code

As previously reported, the tubulin code can modulate intrinsic properties of MTs, such as structural [8,17,18], mechanical [44,45,46] and dynamic features [19,21]. However, its major function is to regulate, in a selective manner, the recruitment of proteins.

Molecular motors are undoubtedly the main readers of the tubulin code. They are a subclass of MAP which use the energy of ATP hydrolysis to walk along MTs and include kinesins, which move towards MT plus-ends and are responsible for the anterograde transport and dyneins, which instead move toward MT minus-ends achieving the retrograde transport [117].

Recently, using a yeast expression system, Sirajuddin and colleagues have shown that the selective incorporation of tubulins into MTs and their PTMs regulates molecular motor velocity and processivity in vitro, with substantial changes conferred by even single amino acid variations among tubulins [118]. The authors demonstrated that the presence or the absence of the C-terminal Y residue on the α-tubulin affects the processivity of Kinesin-1 and Kinesin-2 and the depolymerizing activity of Kinesin-13. Moreover, they showed that the incorporation of TUBB3 or TUBB1 into in vitro-assembled MTs substantially reduces the run length of Kinesin-1. Interestingly, TUBB1 and TUBB3 are the only β-tubulin isotypes that possess a positively charged lysine residue in the C-terminal domain and the removal of this amino acid or the polyglutamylation of the C-terminal tail on TUBB3, which increases its negative charge, fully restores Kinesin-1 motility in vitro [118].

These results have demonstrated that the selective expression of tubulins and the accumulation of PTMs on them can provide specific “signatures” that are recognized only by a restricted set of proteins.

Unfortunately, very little is known about the impact that each single tubulin isotype has on the recruitment of molecular motors, whereas the effect of tubulin PTMs has been mostly characterized in the last few years.

Kinesin-1, for example, preferentially associates with detyrosinated MTs (Figure 2) in different in vitro neuronal models [100,119]. By contrast, Kinesin-5, preferentially associates with tyrosinated MTs in dendrites [120] (Figure 2).

Dynein processivity and activity, instead, have been reported to be mainly regulated by tubulin tyrosination and glutamylation [121,122,123]. The DDB complex, composed of dynein, dynactin and BicD2 protein, selectively binds tyrosinated MTs to initiate its movement (Figure 2), since the motility of the motor complex was significantly reduced on MTs containing recombinant α-tubulins deleted of their C-terminal Y [123]. Moreover, alteration of tubulin glutamylation affects flagellar dynein activity and causes a severe deficiency in ciliary motility associated with an abnormal waveform and reduced beat frequency in *Tetrahymena* [121,122].

**Figure 3:**
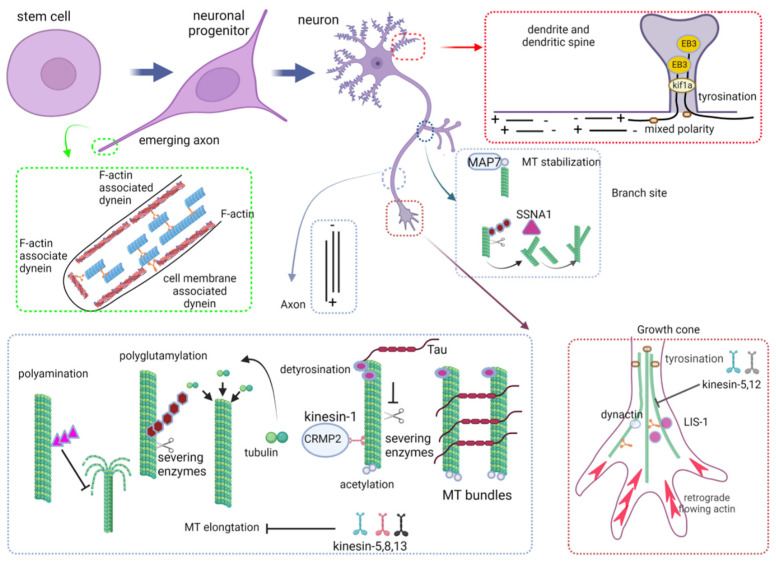
MTs, their PTMs and interactor during neuronal morphogenesis. During axon initiation, MTs are pushed forward *en masse* by dyneins. MT massive translocation generates, on the cell membrane, the mechanical force underlying axon protrusion. In axon, MTs work like a structural backbone to support the axonal compartment. They are organized into aligned, densely packed bundles with their plus-end pointing away from the cell body that is stabilized by Tau. Tau also prevents MT fragmentation catalyzed by the MT-severing proteins katanin and spastin, which are recruited on glutamylated MTs. MTs act also as filters for the passage of molecular motors with axon-specific cargoes, such as Kinesin 1, which preferentially binds detyrosinated and acetylated MTs and transports the CRMP2–tubulin complex, thus promoting the incorporation of free tubulins into the ends of existing MTs. Tubulin polyamination also accumulates into the axon and protects MTs from depolymerization. Kinesin-5, 8 and 13 instead regulate axogenesis by limiting axonal growth. During growth cone pathfinding cytoplasmatic dyneins, dynactin and LIS1 promote MT advance into the growth cone, resisting buckling and collapsing caused by actin cytoskeleton. Kinesin 5 and 12, instead counterbalance the forces generated by cytoplasmic dyneins, opposing MT entry into the growth cone. During axon branching, short MTs are generated through the action of MT-severing enzymes katanin and spastin at newly forming branches and remodeled into branched structures by SSNA1. Axonal branch formation is also promoted by MAP7 which binds the acetylated and stable region of individual MTs, avoiding their dynamic plus-ends and preventing branch retraction. In dendrites, MTs have bidirectional orientation. Dendritic spines are transiently invaded by labile tyrosinated MTs which allow the Kif1A-mediated transport of synaptotagmin 4. The figure has been created in “BioRender.com”.

Tubulin glutamylation also influences the motility of Kif1A (Figure 3), a member of the Kinesin 3 family involved in the axonal transport of synaptic and dense core vesicles. Kif1a interacts with the tubulin C-terminal domain through its positively charged K-loop, located in its motor domain [65]. The lack of polyglutamylated chains on the C-terminal domain of α- and β-tubulins reduces Kif1A pausing and overall run length in vitro [65]. Moreover, hippocampal neurons of ROSA22 mice, that exhibit a striking loss of polyglutamylated α-tubulins, display improper Kif1A localization both in vitro and in vivo, and insufficient cargo delivery of synaptic vesicles in vivo [124].

Another two important readers of the tubulin code are structural MAPs and +TIPs [125]. Structural MAPs are a heterogenous group of proteins that statically bind along the length of MTs, contributing to their stability [126]. They play a critical role in neuronal cells, where they principally contribute to maintaining a polarized and mutually exclusive distribution of MTs in dendrites and axons [127]. These proteins bind MTs with different affinity, depending on their PTM state. The best example of this regulation is provided by tubulin polyglutamylation, which has been reported to regulate MAP transition during neuronal development [125]. In vitro experiments have shown that neuronal MAPs bind with different affinity purified brain tubulins, depending on the length of E chains on their C-terminal domain. MAP1A efficiently binds hyperglutamylated tubulins, while Tau and MAP2 preferentially bind tubulins with moderate levels of polyglutamylation [128] (Figure 2). 

Interestingly, MAP2, which is implicated in the generation of dendrites, preferentially binds glutamylated β-tubulin whose levels accumulate in dendritic compartments during dendritogenesis [128]. Instead, both MAP1A and polyglutamylated β-tubulin increase in dendrites during the second week of post-natal brain development [128]. This evidence suggests that levels of tubulin glutamylation, the length of the glutamylated chain, and the tubulin isotype that is glutamylated (α *versus* β) are the main molecular determinants that regulate the transition of MAPs–tubulin interaction during nervous system development.

Lastly, +TIPs are a subclass of MAP that transiently bind the plus-ends of growing MTs and locally control MT assembly and their attachment to other cellular components such as the cell membrane or kinetochore [129].

It has also been reported that TIPs containing the Cap-G-rich MT-binding domain, such as the cytoplasmic linker protein 170 (CLIP-170), the cytoplasmic linker protein 115 (CLIP-115), and p150 Glued, bind more efficiently tubulins in their tyrosinated state [101,130,131] (Figure 2).

In cultured hippocampal neurons of *TTL*-null mice, which display high levels of detyrosinated tubulin, CLIP-170 has been shown to be misallocated from the distal part of neurites and from growth cones [104]. Moreover, in *S. cerevisiae*, where the detyrosination of α-tubulin does not occur, the removal of the C-terminal aromatic residue of α-tubulin impairs the interaction between MTs and CLIP-170 yeast ortholog, suggesting a crucial role of the C-terminal Y residue for CLIP-170 association with MT plus tips [132].

A list of MT-interacting proteins and tubulin PTMs that enhance and inhibit their MT binding is summarized in Table 2.

## 4. Regulation of MTs in Neurons

Nervous system development and homeostasis depend on the correct functioning of MT cytoskeleton. Developing neurons rely, in fact, on the dynamic nature of MT cytoskeleton to remodel their shape and create complex structures such as axon, dendrites and synapses [151,152,153]. MTs are also key effectors of correct neuronal placement as they provide the traction force that allows newly formed neurons to migrate towards different brain regions. Moreover, MTs support neuronal circuits through the transport of vesicles, proteins and organelles along the axons.

In recent years, a growing number of mutations in genes encoding for tubulins, MAPs and enzymes that post-translationally modify MTs, which are the main factors of the tubulin code, have been linked to neurodegenerative processes or defects in neuronal migration, differentiation and connectivity. However, how the information necessary to coordinate MT functions is established and deciphered during neuronal development is still largely unchartered.

In this section, we will provide an overview of how MTs, their PTMs and interactors can regulate critical neuronal events, such as neuron maturation, migration and axonal transport.

### 4.1. Neuron Maturation

#### 4.1.1. Axon Formation

During neuron maturation, neuronal stem cells undergo dramatic morphological changes that lead to the formation of multiple processes which start as small buds on the membrane and elongate to form thin protrusions, the neurites. Importantly, only one process will mature in the axon while the other will generate the dendrites [154]. 

Axon formation marks the initial morphological event that defines neuronal polarization and largely depends on the local reorganization of MT cytoskeleton [155,156]. Electron micrograph studies have revealed that during in vitro axonogenesis, the cell body of neuronal precursors is gradually depleted of MTs while there is a massive increase in MTs in the neurite that will generate the axon [157].

MT translocation into the nascent axon is mainly driven by dynein motors that push MT meshwork forward *en masse* and generate, on the cell membrane, the mechanical force underlying axon protrusion [158]. In the model proposed by Roossien and colleagues [158], dyneins targeted to actin at the cell cortex or bound to the plasma membrane capture the plus-ends of MTs and use their minus-end activity to move them forward. Furthermore, dyneins bound to two neighbors within a MT overlap produce the force to slide them relative to each other, thus contributing to the displacement of MTs forward along the axon [158]. In vitro experiments have shown that MT disruption or dynein depletion causes axon retraction or impaired axon growth [159,160,161,162]. Moreover, dynein inhibition induces MT bulk retraction and increases the tensile force of the axon in cultured neurons of chick and of the sea slug *Aplysia californica* [163].

The plus-end motor Kinesin1 has also been recently reported to promote axon outgrowth in cultured neurons of *Drosophila*, by sliding MTs against each other and thus forcing membrane extension at the tip of the neurite [135].

In the axon, MTs are aligned into densely packed bundles with their plus-ends pointing toward the tip of the axon [164,165]. This configuration is not fortuitous; its establishment and permanent maintenance requires several MT regulators, primarily MAPs, which impose structural order, thus preventing MTs to go into disarray.

Tau undoubtedly has a pivotal role in MT bundling in the axon. Using a centrosome-mediated MT regrowth assay, Brandt and Lee showed that Tau promotes both MT nucleation and growth in vitro [140]. Recently, Hernandez-Vega and colleagues have demonstrated that under the condition of molecular crowding, Tau forms liquid-like drops in which tubulin is condensed, and drives the nucleation and the elongation of MT bundles [143]. Moreover, Méphon-Gaspard et al. (2016) have reported that Tau mediates, under macromolecular crowding conditions, MT bundle architecture, forming long-range cross-bridges between MTs [142]. Tau’s ability to form MT fascicles would depend on its phosphorylation state. Recently, Rodríguez-Martin et al. (2013) have proposed that the excessive Tau phosphorylation suppresses MT bundle formation in transfected CHO cells [166]; interestingly, mutation affecting Tau’s phosphorylation state have been reported to increase MT spacing in neurites of cultured PC12 cells and in processes of Sf9 cells [167].

Tau also counteracts the activity of the MT-severing proteins katanin and spastin by preventing MT fragmentation in the emerging axon of cultured neurons [141]. The suppression of Tau in cerebellar macroneurons curtails the transformation of the immature neurite into the axon [168], while its induction in NGF-treated PC12 increases MT mass by promoting MT stability and formation [169]. 

Besides working like structural backbones, axonal MTs act also as filters for the passage of molecular motors with axon-specific cargoes. They indeed mobilize, *via* the plus-end-directed transport of kinesin motors, polarity signals required for axon outgrowth. The CRMP2–tubulin complex, which catalyzes the incorporation of free tubulins into the ends of existing MTs, is transported to the distal part of the axon by Kinesin-1 [170,171] while phosphatidylinositol-triphosphate, a factor involved in the spatial restriction of axonogenesis signals, is transported by the Kinesin-3 motor protein GAKIN/Kif13B [172].

Additional kinesins, such as the mitotic Kinesin-5, 8 and 13, instead, have been reported to regulate axonogenesis by limiting axonal growth. Pharmacological inhibition of Kinesin-5 in cultured neurons, for example, results in longer axons [173,174]. Instead, Kinesin-8 and Kinesin-13 inhibit axonal growth by enhancing dynamic instability and promoting MT catastrophe [175,176]. It follows that axonogenesis is a tightly regulated process that needs the interplay of different factors that, if, on one hand, promote axon extension, on the other, counterbalance its excessive growth.

To break neuron symmetry and specify axon formation, MTs also need to be stabilized [177].

MTs were found to be more stable in the axon shaft compared with those of future dendrites [107,177]. Indeed, the ratio of stable (detyrosinated and acetylated) to dynamic MTs (tyrosinated) has been shown to be significantly higher in the axon compartment of hippocampal neurons of stage 2 [177], suggesting that in morphologically unpolarized cells, MT stabilization in one neurite precedes axon formation [177,178]. Accordingly, neurons treated with low doses of Taxol extend axon-like neurites that display a high ratio of acetylated to tyrosinated tubulin [177]. The local stabilization of MTs may also explain the selective targeting, in the nascent axon, of kinesin motors such as Kinesin-1 and GAKIN/Kif13B which preferentially bind acetylated and detyrosinated MTs [133,179], and are critical for neuronal polarization [172].

#### 4.1.2. Growth Cone Formation

A critical step that occurs during axon extension is the formation, at the tip of the axon, of the growth cone [180], a specialized motile structure responsible for directing axon guidance. MTs have been classically considered second players in growth cone pathfinding. Indeed, despite it being well-established that MTs, by extending into the growth cone, are able to drive its turning, their polar invasion has been deemed to passively follow the change in actin organization.

Several pieces of evidence support the existence of mechanisms by which MTs are subjected to forces generated by molecular motors, primarily dyneins, which allow them to invade the growth cone, resisting to buckling and collapsing caused by the actin cytoskeleton.

Recently, Grabham and colleagues have demonstrated that dynein and its regulatory factors LIS1 and dynactin promote MT advance during the growth cone remodeling of cultured chick sensory neurons, and that dynein or LIS1 inhibition reduces the number of MTs invading the peripheral domain of the growth cone and the ability of MT to resist retrograde actin flow [162]. The authors also showed that dynein, LIS1 and dynactin accumulate in the axonal growth cone of cultured hippocampal neurons at stage 3 and that the *LIS1* silencing compromises, in these cells, growth cone organization and axon elongations [162]. Differently, the motor proteins Kinesin 5 and Kinesin 12 have been reported to counterbalance the forces generated by cytoplasmic dyneins, opposing MT entry into the growth cone [181,182,183]. Their depletion in cultured neurons causes a faster-growing axon [181,182] and prevents growth cones from turning properly enhancing MT invasion into filopodia. Moreover, *Kinesin-12* knockdown also results in notably longer fast-growing axons in zebrafish embryos as well [183].

Recently, in vitro evidences have shown that the Kinesin-2 Family Member Kif3C is likewise implicated in the regulation of MT dynamics and organization at the growth cone of DRG neurons, by acting as a MT-destabilizing factor. Kif3C preferentially binds tyrosinated MTs and interacts with the end binding protein (EB) 3, promoting its localization at MT plus-ends. The depletion of this kinesin in adult neurons leads to an increase in stable, overgrown and looped MTs in vitro because of a strong decrease in their frequency of catastrophes [149].

Thus, proper growth cone pathfinding requires the synergic action of different molecular motors, which tune the MT cytoskeleton, by regulating its invasion and organization at the tip of the axon.

In support of a non-marginal role of MTs in growth cone pathfinding and morphogenesis, functional studies have shown that organotypic cultures of the hindbrain obtained from *TTL*^−/−^ mouse embryos, which are deprived of tyrosinated tubulin, have a hypertrophic growth cone with randomly oriented filopodia [184]. Moreover, axons of precerebellar nuclei neurons, in which tubulin detyrosination does not occur, emit supernumerary neurites ex vivo and in vitro. These neurons also present reduced axon length and their axon trajectories are not straight when they grow in a collagen matrix, or ex vivo when they approach the floor plate [184].

#### 4.1.3. Axon Branching

During maturation, the axon develops multiple collateral branches which are located far from the soma and connect with multiple synaptic targets [185,186]. The stabilization of premature branches requires the insertion of axonal MTs [187,188]. Recent studies have demonstrated that, in cultured chick sensory neurons, as well as in rodent hippocampal and cortical neurons, short MTs are transported to or synthesized at newly forming branches [189,190], suggesting that MT fragmentation at localized areas of the axon shaft would increase the amount of available tubulin molecules or short MT fragments that insert into filopodia and promote branch formation [191]. Accordingly, the over-expression of the MT-severing enzyme spastin, which results in a large number of short axonal MTs, dramatically enhances the formation of axonal branches in vitro [192], while the stabilization of the MT cytoskeleton using the MT-stabilizing drug Taxol reduces branch formation by decreasing the invasion of MTs into early filopodia in cultured neurons [187].

Different MAPs promote axonal branch formation [189]. MAP7 has been found to co-localize with MTs at branch junctions of cultured DRG sensory neurons [193,194]. Tymanskyi and Ma (2019) have demonstrated that MAP7 binds the acetylated and stable region of individual MTs, avoiding their dynamic plus-ends and preventing MT depolymerization and branch retraction after laser-induced severing or nocodazole treatment [195]. Moreover, over-expression of MAP7 in younger neurons that do not typically express it increases the number of interstitial branches, while the perturbation of its expression in older neurons, cultured after branch formation in vivo, results in fewer interstitial branches [193].

So far, SSNA1 protein, a MAP with tubulin nucleation activity, has been reported to accumulate in vitro at the branch sites of developing neurons and to enhance the branching patterns of the growing axon [196,197]. Recently, Basnet and colleagues have demonstrated that SSNA1 directly remodels MTs into branched structures by connecting the ends of existing MTs or by splitting the MT lattice [197].

Because this MAP interacts with spastin [146], which localizes at branch sites as well [194], it has been proposed that, at the branch points of the axon, spastin-severed MTs may act as building blocks for SSNA1-mediated MT nucleation and branching. According to this model, SSNA1 mutants that abolish MT branching in vitro also fail to promote axon branching when over-expressed in neurons [197]. 

#### 4.1.4. Dendrite Formation

The last stage of neuron maturation is the acquisition of a proper dendritic architecture and the maturation of dendritic spines, protrusions on dendritic shafts, which are the sites where excitatory synapses are located [198,199].

Differently from axons in which MTs are oriented in a plus-end distal orientation, dendrites contain MTs with both polarities [200]. The establishment of MT mixed polarities in the dendrite occurs earliest during neurite formation [201,202,203], and is achieved through the transport of MTs and through their nucleation at non-centrosomal sites into the developing dendrite.

Kinesin 6 (also known CHO/MKLP1) has been reported to transport minus-end MTs into nascent dendrites. In Kinesin-6-depleted cultured neurons, minus-end MTs fail to move into dendrites and these processes do not acquire their dendritic characteristics [204,205]. Similarly, the silencing of Kinesin-12 decreases the proportion of minus-end-distal MTs in experimental dendrites and impairs their morphology [206].

Furthermore, different works have demonstrated that in neurons, nucleating factors localize into dendrites and drive the nucleation of MTs, both anterogradely and retrogradely, depending on their position and orientation into the dendritic arbor [207,208,209,210].

In the neurons of *C. elegans*, γ-tubulin ring complexes have been found to localize at the growth cone of dendrites and produce both anterograde- and retrograde-polymerizing MT populations, thus creating plus-ends out and minus-ends out of MTs, respectively [209]. MT-organizing centers (MTOCs) have been observed also at the tip of dendrites in *Drosophila* sensory neurons [210]. In vivo time lapse experiments have shown that in this class of neurons, anterograde-polymerizing MTs nucleated at MTOCs are guided into nascent branches to split the tip into new primary branches [210]. MT nucleation in dendrites can occur also on pre-existing MT fragments. In cultured rodent hippocampal neurons, augmin has been demonstrated to recruit γ-tubulin on MT fragments to amplify the existing array [207,208].

Several studies have shown that the regulation of MT cytoskeleton is critical for the appropriate arborization of neuronal dendrites during development. In vitro studies have demonstrated that in sympathetic neurons, activity-dependent dendrite formation is accompanied by the increased association of MAP2 with MTs and increased MT stability [211]. Moreover, Getz and colleagues have reported that Phosphatase and tensin homolog-deficient neurons, which present supernumerary dendritic processes, have increased MT polymerization rates in dendritic growth cones, and that the correction of tubulin polymerization by MT pharmacological inhibition is sufficient to reduce dendritic overgrowth both in vitro and in vivo [212].

Recently, Delandre and colleagues have shown that specialized dendrites of class I and class IV neurons of *Drosophila* differ in MT configuration and density, according to their different sensory modalities [213]. This evidence suggests that MT architectural organization may act as a discriminating factor in establishing neuron-type specific morphologies by regulating dendrite requirements.

The presence of MTs in spines, as well as their role in spine plasticity and morphology, has remained unexplored for a long time. For several years, in fact, it has been believed that only the actin resides in dendritic spines controlling their shape and function and, only recently, MTs have emerged as equally relevant temporal and spatial regulators.

Studies of electron microscopy and live confocal imaging have demonstrated that labile populations of MTs localize into dendritic spines in vitro [214,215], and affinity chromatography experiments have identified a soluble form of tubulins among binding partners of the synaptic NMDA receptor [216,217]. In addition, tubulin mRNA was found in synaptosomal preparations enriched for dendritic spines [218].

Moreover, in vitro experiments have demonstrated that MT manipulations by pharmacological drugs, such as nocodazole and Taxol, modulate the spine development of rat hippocampal neurons [214].

MTs only transiently stay in the spines of cultured neurons [219,220,221]; these populations are mainly tyrosinated and their invasion into dendritic protrusions appears to be dependent on neuronal activity, since the stimulation of neurons with high concentrations of KCl enhances in vitro both the number of spines invaded by MTs and the duration of these invasions [219]. This experimental evidence suggests that the MT cytoskeleton may play an important role in synapse function and plasticity [151,219,220,221]. Accordingly, recent works have shown that MTs support the channel activity of the transient receptor potential family mechanotransduction channel NompC by conveying force to gated channel activation [222,223].

Furthermore, MTs would also be critical for the transport of material throughout the dendritic arbor. Recently, McVicker and colleagues have reported that in cultured neurons, synaptotagmin 4, a vesicular calcium-binding protein implicated in retrograde signaling at synapses, is directly transported into the spine along dynamic MTs by the motor protein Kif1a [224].

A schematic overview of MT, tubulin PTMs and MT-interacting protein tasks during neuronal maturation is illustrated in Figure 2, while a list of MT-interacting proteins and tubulin PTMs involved in neuronal maturation is summarized in Table 3.

### 4.2. Axonal Transport

A key role of the neuronal MT cytoskeleton is to support long-range bidirectional transport, providing lines for molecular motors [226].

MTs do not only have a passive role acting as highways for the movement of motor proteins; efficient and well-orchestrated transport depends on the structural and chemical features of MTs, and on its interaction with MAPs, to guarantee that different cargoes are moved toward specific locations in a finely regulated manner.

Pioneering work in worm neurons has revealed that the length and density of MTs set the upper rate limit of efficient transport [227].

Firstly, the movement of molecular motors and, thus, the duration of cargo transport is limited by the MT length. In vivo experiments have shown that in neurons of mutant strains of *C. elegans*, which harbor shorter or longer axonal MTs, the run length of synaptic vesicle precursors decreases or increases depending on the MT lengths [227].

Moreover, the coverage and the vicinity of MT tip influence the speed of axonal transport and the duration of the immobile state of molecular motors. Recently, Yogev and colleagues, using a novel light-microscopy-based method, have demonstrated that in neurons of live worms, motor proteins stop at the MT ends and that the pause time that they spent in the immobile state is less in strains with higher MT coverage and longer in strains with lower MT density. Similarly, in the distal axon of L4 larvae of *C. elegans*, where the density of MT arrays is lower, the pause times of synaptic vesicles are longer as compared to those of the proximal axon, where MT coverage is significant [227]. In accordance with these observations, Buscaglia and colleagues have recently reported that in cultured cortical neurons of *Tuba1a*^ND/+^mice, harboring the heterozygous p.N102D substitution in Tuba1a that impairs Tuba1a stability and MT thickness, the inappropriate density of MTs causes a lengthening of lysosome stalling in the axon [228].

The resumption of axonal transport, after pausing, depends on the abundance of MTs near the tubulin tips, which allows molecular motors to switch on them and continue the run [227]. 

While structural features of MTs regulate physical parameters of axonal transport, the chemical composition of MTs (tubulin isotypes and PTMs), as well as their interaction with specific MAPs, mainly influences the type of cargo that will be transported and the balance and the distribution of motor proteins.

Recent studies have documented the selective transport of cargoes on specific subsets of modified MTs in neurons in both a physiological and pathological context. In injured dorsal root ganglion cultured neurons, for example, the retrograde transport of lysosomes occurs on tyrosinated MTs, and the knockdown of *TTL*, which decreases the levels of tubulin tyrosination, causes a reduction in the fraction of retrograde-moving lysosomes with a concomitant increase in their stationary state [39]. The efficiency of mitochondrial transport, instead, seems to be selectively regulated by tubulin detyrosination. In the SMN-depleted motoneuron-like NSC34 cell line, an in vitro model of spinal muscular atrophy, which shows a significant down-regulation of α-tubulin detyrosination, mitochondria are misallocated along neurites and their location is restored only after MAP1B depletion that increases the level of detyrosinated α-tubulin [229].

However, among tubulin PTMs, polyglutamylation has attracted great attention in recent years as an active regulator of neuronal trafficking. It has been reported that distinct patterns of glutamylation on α- and β-tubulins influence axonal transport [9,67]. Different works have demonstrated that excessive tubulin glutamylation strongly decreases mitochondria motility in cultured hippocampal pyramidal neurons and cerebellar granule cells (CGC) [93,230,231]. Moreover, in neurons of *pcd* mouse, which present high levels of glutamylated α- and β-tubulins, only the loss of TTL1, the enzyme that polyglutamylates α-tubulin, leads to increased mitochondria motility, while the loss of TTLL7, that polyglutamylates β-tubulin, has no such effect [67]. This evidence demonstrates that motor proteins have different sensitivity to the type of tubulin that is glutamylated (α *versus* β).

The hyperglutamylation of tubulin has been also reported to reduce, in cultured hippocampal neurons, the motility and the run length of a wide range of functionally different neuronal cargoes, such as lysosomes, LAMP1, endosomes and BDNF vesicles [230], thus suggesting that this tubulin PTM, more than the others, may act as a general traffic regulator in neurons.

Tubulin PTMs can also influence the biophysical parameters of cargo transport, despite, in some cases, their effect being relatively modest: detyrosination decreases the motility of the activated DDB complex 4-fold [123], acetylation increases Kinesin-1 velocity 1.2-fold [232], and detyrosination decreases Kinesin-1 processivity 1.4-fold [118].

The interaction between motors and nonenzymatic MAPs is another emerging regulatory mechanism of cargo transport. A recent systematic analysis of the effects of MAPs on the transport mediated by different molecular motors revealed the distinct influences of MAP7 and MAP9 on Kinesin-1, Kinesin-3 and dynein motor movements [233]. In this study, Monroy and colleagues, by using in vitro reconstitution experiments of purified proteins, demonstrated that MAP7 exerts a dual-opposite effect depending on the type of motor proteins, promoting the recruitment of Kinesin-1 and inhibiting the binding of Kinesin 3 on the MT lattice [233]. The authors also showed that MAP9 enhances the binding and motility of Kinesin3 on MTs and, at the same time, inhibits the processive dynein–dynactin complex from accessing the lattice [233]. Overall, this work provides evidence that MAPs can regulate the distribution of molecular motors by gating access to the MT network.

MAPs can also compete for the binding of MTs, as in the case of MAP7 and Tau, which differently regulate Kinesin-1 motility in vitro, on MT assemblied from purified tubulins, and, in vivo, in *Drosophila* peripheral nervous system neurons; this competition dictates the access of Kinesin-1 to MTs determining its correct distribution and the balance of its motor activity [234].

While the role of MAP and tubulin PTMs have been well-characterized over the years, very little or almost nothing is known about the contribution that each neuronal tubulin has on the regulation of the critical parameters of axonal transport or, rather, if MTs composed of specific isotypes may be used as preferential binaries by molecular motors. 

The recent evidence that mutations in specific neuronal tubulins, such as TUBB3 and TUBB2A which preferentially affect axonal transport by disrupting the binding of specific motor proteins and are associated with the loss of axons in the central nervous system and peripheral neuropathy [23,235], may suggest that MTs composed of these isoforms could act as preferential tracks for these motor proteins in neurons. However, most of these mutations affect highly conserved and adjacent residues located at the motor interface of tubulins, whose negative charge has been demonstrated to be critical for the kinesin binding and stall force [235,236,237]. In addition, Niwa and colleagues have demonstrated that these variants exert the same pathogenic effects when introduced in other tubulin isotypes and then overexpressed in neurons [237]. Therefore, specific amino acids on tubulins, rather than specific tubulins, are critical for the proper axonal transport in neurons.

However, a recent work has demonstrated that in worm neurons, the α-tubulin TBA-6 regulates the velocity and the type of cargoes of intraflagellar transport mediated by Kinesin-2 and OSM-3/Kif17 motor proteins in vivo [238]. So, although currently there is no evidence to support it, it cannot be excluded that also in other contexts, as in axonal transport, specific tubulins may determine the hallmarks and physical parameters of cargo transport.

MTs supporting axonal transport travel through the axon initial segment and the transport of substances occurs from the minus-end toward the plus-end of the MTs, ending at the axon tip. Importantly, the EB protein family (composed of EB1, EB2 and EB3), is of primary importance in coordinating proteins on the growing MT plus-ends and, in fact, they are also described as +TIPS. In particular, EB3 accumulates and stabilizes the axon initial segment of mature neurons and it is used to quantify different features of axonal transport by transfection of GFP-EB3 plasmids that allow visualizing and measuring the speed and directionality of EB3-positive comets present in the growing MT plus-end [239].

### 4.3. Neuronal Migration

For the proper assembly of neural circuits, newly born neurons must migrate from their place of origin to their final location within the central nervous system. This event occurs during development and is maintained in specific areas in the adult brain [240]. It consists of the repetition of three synchronized steps:

(i). The extension of a large cell protrusion known as the leading process;
(ii). The translocation of the nucleus into the leading process. This event is preceded by the formation, at the proximal region of the leading process, of a cytoplasmic dilation known also as swelling;
(iii). The retraction of the trailing process that allows the cell body to move forward in the direction of migration [241].

Perturbations in these sequential steps generate in humans cortical malformations such as lissencephaly, a set of rare brain disorders whereby the whole (agyria) or parts (pachigyria) of the surface of the brain appear smooth [242] and polymicrogyria, a condition characterized by the presence of many and smaller ridges or folds in the brain [243].

Neurons rely on the MT cytoskeleton to produce the traction force for the extension of the leading process and for soma translocation. 

**Figure 4:**
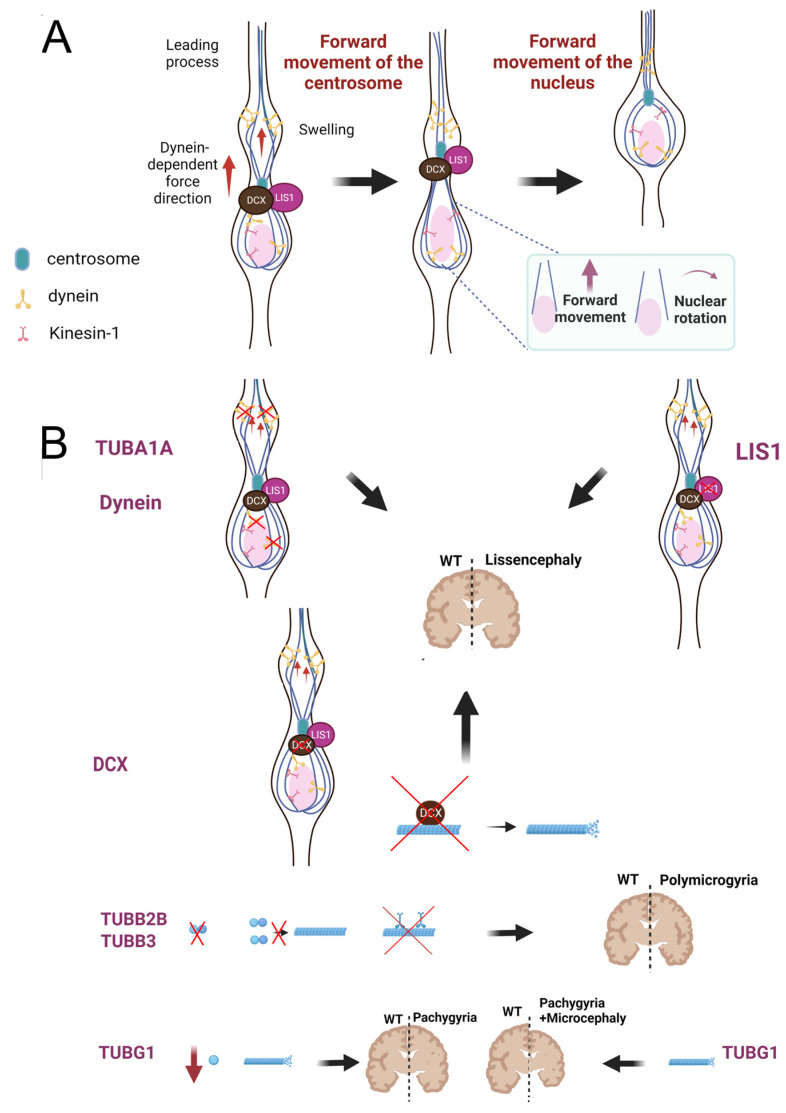
Overview of MT tasks during neuronal migration. (**A**) During neuronal migration, MTs radiate from the centrosome to extend anteriorly into the leading process and posteriorly around the nucleus, forming a cage structure that surrounds the nucleus. Dyneins are enriched at the swelling of the leading process, where they interact with the plus-end of MTs attached to the centrosome, pulling them forward using their minus-end directed activity. The centrosome is dragged by the dynein-dependent force into the swelling region; then, the nucleus funnels through the leading process and catches up with the centrosome. Dyneins and Kinesin1 are also connected to the nuclear envelope and, by exerting a transient MT-dependent force, steer nuclear rotation and forward translocation into the leading process. LIS1 and Doublecortin (DCX), instead, localize at the centrosome region and regulate the coupling of the nucleus to the centrosome. (**B**) In humans, mutations affecting the arginine (R) in position 402 of TUBA1A, dyneins and LIS1 disrupt the activity of cytoplasmic dyneins causing lissencephaly. Mutations in DCX instead impair MT stabilization and structure causing the lissencephalic phenotype. The majority of *TUBB2B* mutations and a small number of *TUBB3* mutations are associated with polymicrogyria. These variants impair the formation of α/β-tubulin heterodimers or their incorporation into MTs. TUBG1 mutations affect MT dynamics and stabilization causing pachygyria in association with microcephaly or laminar heterotopia. The figure has been created in “BioRender.com”.

In CGC migrating in vitro and in ex vivo slice preparation, MTs radiate from the centrosome, extending anteriorly into the leading process and posteriorly around the nucleus, forming a cage structure that surrounds the nucleus [244,245] [Figure 4).

Experiments of fluorescence microscopy have revealed, in locomoting neurons, an enrichment of dynein at the swelling of the leading process, as well as a polar network of parallel MTs emanating from the centrosome with their plus-ends oriented toward the leading process [246]. At the swelling, dyneins are properly positioned to interact with plus-ends of MTs attached to the centrosome [246], raising the possibility that, by using their minus-end-directed activity, dyneins may exert a point of force on MTs, pulling them forward [246]. Under this driving force, the centrosome departs from the nucleus and moves into the swelling region; then, the nucleus funnels through the leading process and catches up with the centrosome (Figure 4). 

According to this model, dynein inhibition or MT disruption by nocodazole treatment impairs the nucleus–centrosome coupling during the migration of cultured neurons [246,247].

Cytoplasmatic dyneins, together with Kinesin-1, have been also reported to be connected, via the LINC complex, to the nuclear envelope of in vitro-migrating CGC [248].

A recent high-resolution time-lapse observation revealed that in this type of neuron, the nucleus undergoes frequent rotation during migration. Moreover, live-cell imaging suggested that MTs in the perinuclear region have mixed polarity and dynamically attach to and detach from the nucleus [248]. This evidence supports the hypothesis that kinesin and dynein motors, by exerting transient forces to multiple points on the nuclear envelope, translocate forward to the nucleus when the net force acts on the center of mass or generates torque and drives nuclear rotation [248].

Consistent with a critical role of MTs and these motor proteins in neuronal migration, Kinesin-1 loss affects normal migration in vivo [245] and defects in the dynein heavy chain, which impair the dynein run length, disrupts neocortical lamination and neuronal migration in mice [249]. Furthermore, blocked MTs reduce nuclear transport during neuronal migration in vitro and abolish the movement of neurons throughout the brain [245]. 

In addition, LIS1 and DCX, a member of MAPs47, have been reported to assist nucleokinesis by regulating the coupling of the nucleus to the centrosome [247]. These proteins display adjacent localization in live migrating neurons, with LIS1 localizing mainly to the centrosome and DCX outlining MTs extending from the perinuclear cage intersecting the centrosome [247]. In humans, heterozygous mutations in *LIS1* result in lissencephaly; moreover, mice with decreased levels of *Lis1* exhibit dose-dependent disorganized cortical layers, hippocampus and olfactory bulb because of cell-autonomous neuronal migration defects [250]. 

DCX has also a role in MT stabilization by promoting bundles and suppressing catastrophes against shrinkage [251,252]. Furthermore, DCX influences MT structures and nucleation; indeed, in vitro experiments have demonstrated that MTs polymerized with DCX are composed of 13 protofilaments while those polymerized in the absence of DCX varied from 8 to 19 [253].

Mutations in *DCX* prevent neurons from migrating into the cortical plate [252]. Moreover, *Dcx*-deficient neurons have been shown to migrate in a disorganized manner, extending and retracting short branches [254]. *Dcx* deficiency also affects, in in vitro-migrating neurons, nuclear movement, decreasing swelling formation and impairing its dynamics [254].

Mutations in the *DCX* human gene predominantly cause lissencephaly in hemizygous males and subcortical band heterotopia, the ectopic positioning of neurons during cortex development with the formation of ectopic nodules, in heterozygous females [255,256].

A role of specific tubulins, most notably TUBA1A, TUBB2B, TUBB3 and TUBG1, have also been reported in neuronal migration [257,258,259,260,261].

*TUBA1A* is the most commonly affected gene in tubulinopathic patients. To date, more than 40 substitutions in the *TUBA1A* gene have been identified in humans and the majority of them cause the lissencephalic phenotype [262,263,264].

*TUBA1A* mutations affecting the R402 account for 30% of all reported mutations [261]. Structural analysis has shown that R402 amino acid is located close to the C-terminal of α-tubulin [265], and functional studies have reported that this residue is critical for the binding and the activity of multiple MAPs and molecular motors including dyneins, VAPB, REEP1, EZRIN and PRNP [261,266].

Recently, Aiken and colleagues have shown that R402C and R402H substitutions dominantly disrupt cortical neuronal migration during mouse brain development [261]. The authors demonstrated that R402 mutant tubulins fail to support the activity of dyneins in yeast and that, in these cells, the level of dynein impairment scales with the expression of R402 mutant tubulins, suggesting a “poisoning” mechanism in which mutant tubulins act dominantly by populating MTs [261].

Differently, the S140G missense mutation in Tuba1a identified in the hyperactive N-ethyl-N-nitrosourea-induced mutant mouse, which presents abnormalities in the laminar architecture of the hippocampus and cortex [267], impairs the speed and the direction during neuronal saltatory migration in vitro by affecting the conformation of α/β heterodimers and increasing the straightness of newly polymerized MTs [268]. The increase in MT straightness has been proposed to affect the nucleus-centrosome coupling during migration and to make MTs more sensitive to mechanical stress, potentially contributing to excessive neuronal branching.

The majority of *TUBB2B* mutations, and a small number of *TUBB3* mutations, instead, has been associated with polymicrogyria [259], a malformation of cortical development in which the brain surface is irregular and the normal gyral pattern is replaced by an excessive number of small and partly fused gyri separated by shallow sulci [269].

Most of the *TUBB2B* mutations related to polymicrogyria impair the ability to form functional α/β-tubulin heterodimers [270]. Two mutations (E421K and D417N) have been reported to impair MT binding with motor proteins [271,272]; interestingly, the p.E421K substitution also causes congenital fibrosis of the extraocular muscles, which is compatible with a primary axonal dysinnervation and with the hypothesis that mutations affecting the binding with molecular motors can drive neurodegeneration as well [271].

Analogously to *TUBB2* mutations, *TUBB3* variants result, in vitro, in an inefficient α/β-tubulin heterodimer formation and in a subtle impairment of mutant TUBB3 ability to incorporate into the cytoskeleton [273].

Mutations in *TUBG1* were identified in patients with pachygyria which can be in association with microcephaly (p.L387P, p.W92C and p. S259L) or laminar heterotopia (p.T331P) [33].

By using in utero electroporation, Ivanova and colleagues overexpressed *TUBG1* variants in the knock-in *Tubg1*^Y92C/+^ mouse model, showing that substitutions in the *TUBG1* gene affect neuronal positioning by disrupting neuronal migration without a major effect on progenitor proliferation [33]. At the molecular and cellular level, the authors also demonstrated that *TUBG1* variants exert their pathogenicity by affecting MT dynamics (documented by evaluating the MT length and velocity of EB3 comets in HeLa cells and in patients’ primary fibroblasts) rather than centrosomal positioning.

Accordingly, in a previous work, Poirier and colleagues [258] showed that the expression of two of the variants (p.W92C and p.L387P) characterized by Ivanova et al. have a predominant impact on nuclear positioning and MT organization and dynamics in yeast cells [258]. Interestingly, at the molecular level, the authors also demonstrated that the p.L387P substitution drastically affects the chaperonin-dependent folding of TUBG1, compromising the yield of monomeric γ-tubulin [258].

In sum, among the previously described disorders related to neuronal migratory defects, the following neurodevelopmental alterations occur:

Cortical gyration disorder, which includes:

− Lissencephaly, consisting of the absence (agyria) or incomplete development of the brain gyri or convolution (pachygyria), causing the brain’s surface to appear unusually smooth and caused by defects in in neuronal positioning and further differentiation. Mutations in *LIS1* and *DCX* account for approximately 85% patients with lissencephaly [274]. Moreover, among tubulinopathies, pathogenic variants in *TUBA1A* have been most frequently associated with lissencephaly.
− Polymicrogyria, a neurological condition that affects the development of the human brain through multiple small gyri (microgyri) creating excessive folding of the brain, leading to an abnormally thick cortex [255,256] and that develops between the late stage of neuronal migration and the early point of cortical organization [275,276]. This condition is predominantly associated with *TUBB2B* mutations and to a small set of *TUBB3* substitution, among tubulinopathies.
− Neuronal heterotopia, which consists of the development of neuronal populations in aberrant locations due to dysfunctional neuronal migration, and is mainly associated with *DCX* and *TUBG1* genes.

## 5. Conclusions

MTs assist all morphogenetic and functional transitions occurring during neuronal development. They provide structural force to support complex neuronal structures and create routes for the transport of cargoes between these compartments. MTs also allow neuronal precursors to reach their place of maturation within the central nervous system, assisting nucleokinesis and allowing the extension of the leading process.

To fulfill all these specialized tasks, neuronal MTs need to be precisely controlled.

MT regulation occurs at multiple levels through the differential incorporation of tubulin isotypes into the MT lattice, their further diversification through the addition of PTMs, and lastly, through their interaction with multiple protein interactors. These modulations, by acting in a combinatorial manner, change as needed, both structural and physical parameters of MTs, adapting them to specific cellular functions.

The disruption of these regulations dramatically affects neuron functionalities and survival. Perturbations of the tubulin code, indeed, have been linked to a wide range of neurological diseases including ciliopathies, tubulinopathies and several neurodegenerative diseases such as Alzheimer’s and Parkinson’s.

In recent years, several therapeutic approaches have aimed to restore the proper functioning of the tubulin code, attempting both in vitro and in vivo models. Nevertheless, despite the advances in this field, the direct effects of the tubulin code on neuron behaviors are still not completely understood. Moreover, the mechanisms by which each drug works is still an area of intense speculation and further studies are necessary to understand their exact mechanisms of action and finely tune their effects on MTs.

Further studies aiming to understand how MTs are modulated during neuronal functions and how the tubulin code is compromised in neuronal diseases are needed: this is crucial to understand MT regulation and function in neuron physiology and physiopathology with the aim to improve current therapeutic strategies and set up innovative ones.

## Figures and Tables

**Table 1:** List of tubulin PTMs.

Modification	Substrate Preference (α *versus* β-tubulin)	Site	Forward Enzyme	Reverse Enzyme	Function	Evolutionary Conserved	Detected in Neuron	Reference
Acetylation	α-tubulin	Lysine (K) 40	α-tubulin acetyltransferase 1 (ATAT1)	HDAC6, Sirt2	Recruitment of motor proteins, inhibition of MT depolymerization, protection of MTs from mechanical breakage	Yes	Yes	[40,41,42,43,44,45,46,47,48]
Acetylation	β-tubulin	K252	San	Unknown	Inhibition of tubulin dimer incorporation into MTs	Unknown	No	[49]
Acetylation	α-tubulin	K394	Unknown	Unknown	Regulation of MT stability	Unknown	Yes	[50]
Detyrosination	α-tubulin	Removal of C-terminal Tyrosine (Y)	Vasohibin (VASH)1 and 2	Tubulin-tyrosine ligase (TTL)	Inhibition of MT depolymerization	Yes	Yes	[51,52,53,54]
Δ2 deglutamylation	α-tubulin	Removal of C-terminal Glutamate (E) of detyrosinated MTs	Cytosolic carboxypeptidases (CCPs)	No reverse reaction known	MT stabilization	Yes	Yes	[54,55,56]
Polyglutamylation	α- and β-tubulin	Addition of γ-linked E to C-terminal	TTL	CCPs	MT-associated protein (MAP) interactions, recruitment of motor proteins, regulation of trafficking, recruitment of MT severing enzymes	Yes	Yes	[55,57,58,59,60,61,62,63,64,65,66,67]
Polyamination	β-tubulin	Glutamine (Q) 15	Transglutaminase (TG) 1, 2, 3, 6	No reverse reaction or enzymes known	MT stabilization form cold-induced depolymerization	Unknown	Yes	[68]
Polyamination	α-tubulin	Q31, Q128, Q133, Q256, Q285	TG1, 2, 3, 6	No reverse reaction or enzymes known	Putative role in MT lattice stabilization	Unknown	Yes	[68,69]
Glycation	α- and β-tubulin	Various	Nonenzymatic PTM	No reverse reaction known	Inhibition of GTP-dependent tubulin polymerization	Unknown	Yes	[70,71]
Phosphorylation	β-tubulin	Serine (S)172	CDK1	Unknown	Inhibition of tubulin dimer incorporation into MTs	Unknown	No	[72]
Phosphorylation	β-tubulin	S172	MNB/DYRK1	Unknown	MT dynamics and dendrite morphogenesis	Unknown	Yes	[73]
Phosphorylation	α-tubulin	Unidentified residue within the carboxy-terminal	Syk	Unknown	Inhibition of tubulin incorporation into MTs	Unknown	No	[74]
Phosphorylation	α- and β-tubulin	Y residues (not identified)	Src	Unknown	Unknown	Unknown	No	[75]
Methylation	α-tubulin	K40	SETD2	Unknown	MT formation	Unknown	Yes	[76,77]
Sumoylation	α-tubulin	Multiple	Unidentified enzymes	Unknown	Reduction in interprotofilament interaction, promotion of MT catastrophe, inhibition of MT polymerization	Unknown	Yes	[78,79,80]
Ubiquitination	α-tubulin	Multiple	Parkin	No reverse reaction or enzymes known	MT degradation	Unknown	No	[81]
Palmitoylation	α-tubulin	Cysteine (C) 376	Unidentified enzymes	No reverse reaction or enzymes known	Interactions with membranes and subcellular trafficking	Unknown	Detected on brain tubulin purified from rat brain	[82,83,84]
Glycylation	α- and β-tubulin	Add γ-linked Glycine (G) to C-terminal E	TTL3, TTL8	No reverse reaction or enzymes known	Assembly of motile cilia and stability and maintenance of axonemes in sperm tails in *Drosophila melanogaster*	Yes	No	[8,85,86,87,88]
Polyglycylation	α- and β-tubulin	Add γ-linked G	TTL10	No reverse reaction or enzymes known	Unknown	Not conserved in humans	No	[85]

**Table 2:** List of MT-interacting protein and tubulin PTMs enhancing and inhibiting their binding to MTs.

MT-Interacting Protein	Neuronal Compartment	Role	Function	Tubulin PTMS Enhancing MT Binding	Tubulin PTMS Inhibiting MT Binding	Reference
Kinesin-1	Axon	Molecular motor	Axonal transport and axon outgrowth	Detyrosination and acetylation	Tyrosination	[100,119,133,134,135,136]
Kinesin-5	Dendrites	Molecular motor	Transport of minus-end distal MTs into dendrites and regulation of dendritic MT polarity orientation	Tyrosination	Detyrosination	[120]
DDB complex	Terminal axon	Molecular motor	Axonal transport	Tyrosination	Detyrosination	[123]
Kif1a	Axon	Molecular motor	Axonal transport	Glutamylation	Deglutamylation to short E side chain	[65]
MAP1A	Dendrites	Structural MAP	Organization and maintenance of the neuronal MT network	Hyperglutamylation	Deglutamylation to shorten E side chain	[128,137]
MAP2	Dendrites	Structural MAP	MT stabilization in dendrites	Moderate levels of polyglutamylation (one to three E units)	High level of polyglutamylation (up to six E units)	[138,139]
Tau	Axon	Structural MAP	MT stabilization, MT bundling, MT protection from severing enzymes	Moderate levels of polyglutamylation (one to three E units)	High level of polyglutamylation (up to six E units)	[138,140,141,142,143]
CLIP-170	Distal neurite and growth cone	TIP	Regulation of MT dynamics	Tyrosination	Detyrosination	[101,130]
CLIP-115	Dendrites	TIP	Organelle translocation	Tyrosination	Detyrosination	[130,144]
p150 Glued	Distal neurite and growth cone	TIP	MT stability and dynein-mediated axonal transport	Tyrosination	Detyrosination	[130,145]
Spastin	Axon and axon branches	Severing enzyme	Control of MT mass, axon branch formation	Long glutamylated side chains	Detyrosination and acetylation	[63,66,146,147,148]
Kif3C	Growth cone	Molecular motor	Regulation of MT dynamics	Tyrosination	Detyrosination	[149]
Katanin	Axon and axon branches	Severing enzyme	Control of MT mass, axon branch formation	Long E side chains	Detyrosination	[66,109,150]

**Table 3:** List of different components of the tubulin code and their function during neuron maturation.

Neuronal Compartment		Component of the Tubulin Code	Function	Reference
Axon	MAPs	Tau	MT bundling and nucleation, protection of MTs from the activity of the MT-severing enzymes.	[141,142,143,225]
	Motor proteins	Dynein	Promotion of the forward movement of MTs in the nascent axon.	[158]
		Kinesin-1	Transport of the CRMP2–tubulin complex and MT sliding.	[135,170,171]
		Kinesin-3	Transport of phosphatidylinositol-triphosphate.	[172]
		Kinesin-5	Suppression of the forward advancement of MT in the nascent axon.	[173,174]
		Kinesin-8	MT instability.	[175]
		Kinesin-13	MT instability.	[176]
	Tubulin PTMs	α-tubulin acetylation	MT stability; recruitment of Kinesin-1 and Kif13B.	[44,45,46,177]
		α-tubulin detyrosination	MT stability; recruitment of Kinesin-1 and Kif13B.	[94,177]
Growth cone	Dynein-associated factor	LIS1	Promotion of MT advancement in the growth cone.	[162]
		Dynactin	Promotion of MT advancement in the growth cone.	[162]
	Motor proteins	Dynein	Promotion of MT advancement in the growth cone.	[162]
		Kinesin-5	Opposition to MT entry into the growth cone.	[181]
		Kinesin-12	Opposition to MT entry into the growth cone.	[183]
		Kinesin-2	Regulation of MT dynamics and organization at the growth cone; recruitment of EB3 at the MT plus-end.	[149]
	Tubulin PTMs	α-tubulin tyrosination	MT instability.	[94]
Axon branch	MAPs	MAP7	MT stabilization.	[193,194,195]
		SSNA1	Remodeling of MTs into branched structures.	[197]
	MT-severing enzymes	Spastin	Generation of MT fragments.	[197]
Dendrite	Motor proteins	Kinesin-6	Transport of minus-end MTs into nascent dendrites.	[204,205]
		Kinesin-12	Transport of minus-end MTs into nascent dendrites.	[206]
	MAPs	MAP2	Neural-activity-dependent dendrite formation.	[211]
Dendritic spine	Tubulin PTMs	α-tubulin tyrosination	MT instability.	[94]
	Motor proteins	Kif1a	Transport of synaptotagmin 4.	[224]

## Abbreviations

MTs	Microtubules
PTMs	Post-translational modifications
+TIPS	MT plus-end-tracking proteins
DRG	Cultured dorsal root ganglion
K	Lysine
ATAT1	α-tubulin acetyltransferase 1
HDAC6	Histone deacetylase 6
Y	Tyrosine
Vasohibin	VASH
TTL	Tubulin tyrosine ligase
E	Glutamate
CCPs	Cytosolic carboxypeptidases
MAP	MT-associated protein
Q	Glutamine
TG	Transglutaminase
S	Serine
C	Cysteine
G	Glycine
+TIPS	Plus-end-tracking proteins
CLIP-170	Cytoplasmic linker protein 170
CLIP-115	Cytoplasmic linker protein 115
EB	End-binding
MTOC	MT-organizing center
DCX	Doublecortin
R	Arginine

## References

[1] Lasser M., Tiber J., Lowery L.A. (2018). The Role of the Microtubule Cytoskeleton in Neurodevelopmental Disorders. Front. Cell. Neurosci..

[2] Akhmanova A., Steinmetz M.O. (2008). Tracking the ends: A dynamic protein network controls the fate of microtubule tips. Nat. Rev. Mol. Cell Biol..

[3] Chaaban S., Brouhard G.J. (2017). A microtubule bestiary: Structural diversity in tubulin polymers. Mol. Biol. Cell.

[4] van Haren J., Wittmann T. (2019). Microtubule Plus End Dynamics—Do We Know How Microtubules Grow?. Cells boost microtubule growth by promoting distinct structural transitions at growing microtubule ends. Bioessays.

[5] Akhmanova A., Steinmetz M.O. (2019). Microtubule minus-end regulation at a glance. J. Cell Sci..

[6] Akhmanova A., Steinmetz M.O. (2015). Control of microtubule organization and dynamics: Two ends in the limelight. Nat. Rev. Mol. Cell Biol..

[7] Janke C., Magiera M.M. (2020). The tubulin code and its role in controlling microtubule properties and functions. Nat. Rev. Mol. Cell Biol..

[8] Gadadhar S., Bodakuntla S., Natarajan K., Janke C. (2017). The tubulin code at a glance. J. Cell Sci..

[9] Bodakuntla S., Jijumon A.S., Villablanca C., Gonzalez-Billault C., Janke C. (2019). Microtubule-Associated Proteins: Structuring the Cytoskeleton. Trends Cell Biol..

[10] Baas P.W., Rao A.N., Matamoros A.J., Leo L. (2016). Stability properties of neuronal microtubules. Cytoskeleton.

[11] Sakakibara A., Ando R., Sapir T., Tanaka T. (2013). Microtubule dynamics in neuronal morphogenesis. Open Biol..

[12] Guedes-Dias P., Holzbaur E.L.F. (2019). Axonal transport: Driving synaptic function. Science.

[13] Kawauchi T. (2017). Tubulin isotype specificity in neuronal migration: Tuba8 can’t fill in for Tuba1a. J. Cell Biol..

[14] Borys F., Joachimiak E., Krawczyk H., Fabczak H. (2020). Intrinsic and Extrinsic Factors Affecting Microtubule Dynamics in Normal and Cancer Cells. Molecules.

[15] Cappelletti G., Cartelli D., Christodoulou M.S., Passarella D. (2017). Microtubule-Directed Therapeutic Strategy for Neurodegenerative Disorders: Starting From the Basis and Looking on the Emergences. Curr. Pharm. Des..

[16] Fulton C., Simpson P. (1976). Selective synthesis and utilization of flagellar tubulin. The multi-tubulin hypothesis. Cell Motil..

[17] Honda Y., Tsuchiya K., Sumiyoshi E., Haruta N., Sugimoto A. (2017). Tubulin isotype substitution revealed that isotype combination modulates microtubule dynamics in C. elegans embryos. J. Cell Sci..

[18] Ti S.C., Alushin G.M., Kapoor T.M. (2018). Human β-Tubulin Isotypes Can Regulate Microtubule Protofilament Number and Stability. Dev. Cell.

[19] Pamula M.C., Ti S.C., Kapoor T.M. (2016). The structured core of human β tubulin confers isotype-specific polymerization properties. J. Cell Biol..

[20] Vemu A., Atherton J., Spector J.O., Szyk A., Moores C.A., Roll-Mecak A. (2016). Structure and Dynamics of Single-isoform Recombinant Neuronal Human Tubulin. J. Biol. Chem..

[21] Vemu A., Atherton J., Spector J.O., Moores C.A., Roll-Mecak A. (2017). Tubulin isoform composition tunes microtubule dynamics. Mol. Biol. Cell..

[22] Latremoliere A., Cheng L., DeLisle M., Wu C., Chew S., Hutchinson E.B., Sheridan A., Alexandre C., Latremoliere F., Sheu S.H. (2018). Neuronal-Specific TUBB3 Is Not Required for Normal Neuronal Function but Is Essential for Timely Axon Regeneration. Cell Rep..

[23] Tischfield M.A., Baris H.N., Wu C., Rudolph G., Van Maldergem L., He W., Chan W.M., Andrews C., Demer J.L., Robertson R.L. (2010). Human TUBB3 mutations perturb microtubule dynamics, kinesin interactions, and axon guidance. Cell.

[24] Panda D., Miller H.P., Banerjee A., Ludueña R.F., Wilson L. (1994). Microtubule dynamics in vitro are regulated by the tubulin isotype composition. Proc. Natl. Acad. Sci. USA.

[25] Miller F.D., Naus C.C., Durand M., Bloom F.E., Milne R.J. (1987). Isotypes of alpha-tubulin are differentially regulated during neuronal maturation. J. Cell Biol..

[26] Gloster A., Wu W., Speelman A., Weiss S., Causing C., Pozniak C., Reynolds B., Chang E., Toma J.G., Miller F.D. (1994). The T alpha 1 alpha-tubulin promoter specifies gene expression as a function of neuronal growth and regeneration in transgenic mice. J. Neurosci..

[27] Gloster A., El-Bizri H., Bamji S.X., Rogers D., Miller F.D. (1999). Early induction of Talpha1 alpha-tubulin transcription in neurons of the developing nervous system. J. Comp. Neurol..

[28] Leandro-García L.J., Leskelä S., Landa I., Montero-Conde C., López-Jiménez E., Letón R., Cascón A., Robledo M., Rodríguez-Antona C. (2010). Tumoral and tissue-specific expression of the major human beta-tubulin isotypes. Cytoskeleton.

[29] Breuss M., Heng J.I., Poirier K., Tian G., Jaglin X.H., Qu Z., Braun A., Gstrein T., Ngo L., Haas M. (2012). Mutations in the β-tubulin gene TUBB5 cause microcephaly with structural brain abnormalities. Cell Rep..

[30] Hersheson J., Mencacci N.E., Davis M., MacDonald N., Trabzuni D., Ryten M., Pittman A., Paudel R., Kara E., Fawcett K. (2013). Mutations in the autoregulatory domain of β-tubulin 4a cause hereditary dystonia. Ann. Neurol..

[31] Rustici G., Kolesnikov N., Brandizi M., Burdett T., Dylag M., Emam I., Farne A., Hastings E., Ison J., Keays M. (2013). ArrayExpress update--trends in database growth and links to data analysis tools. Nucleic Acids Res..

[32] Breuss M., Morandell J., Nimpf S., Gstrein T., Lauwers M., Hochstoeger T., Braun A., Chan K., Sánchez Guajardo E.R., Zhang L. (2015). The Expression of Tubb2b Undergoes a Developmental Transition in Murine Cortical Neurons. J. Comp. Neurol..

[33] Ivanova E.L., Gilet J.G., Sulimenko V., Duchon A., Rudolf G., Runge K., Collins S.C., Asselin L., Broix L., Drouot N. (2019). TUBG1 missense variants underlying cortical malformations disrupt neuronal locomotion and microtubule dynamics but not neurogenesis. Nat. Commun..

[34] Bahi-Buisson N., Maillard C., Adam M.P., Ardinger H.H., Pagon R.A. (2016). Tubulinopathies Overview. GeneReviews.

[35] Hausrat T.J., Radwitz J., Lombino F.L., Breiden P., Kneussel M. (2021). Alpha- and beta-tubulin isotypes are differentially expressed during brain development. Dev. Neurobiol..

[36] Smith B.N., Ticozzi N., Fallini C., Gkazi A.S., Topp S., Kenna K.P., Scotter E.L., Kost J., Keagle P., Miller J.W. (2014). Exome-wide rare variant analysis identifies TUBA4A mutations associated with familial ALS. Neuron.

[37] Bally J.F., Kern D.S., Fearon C., Camargos S., Pereira da Silva-Junior F., Barbosa E.R., Ozelius L.J., de Carvalho Aguiar P., Lang A.E. (2022). DYT-TUBB4A (DYT4 Dystonia): Clinical Anthology of 11 Cases and Systematized Review. Mov. Disord. Clin. Pract..

[38] Preitner N., Quan J., Nowakowski D.W., Hancock M.L., Shi J., Tcherkezian J., Young-Pearse T.L., Flanagan J.G. (2014). APC is an RNA-binding protein, and its interactome provides a link to neural development and microtubule assembly. Cell.

[39] Song Y., Brady S.T. (2015). Post-translational modifications of tubulin: Pathways to functional diversity of microtubules. Trends Cell Biol..

[40] L’Hernault S.W., Rosenbaum J.L. (1985). Chlamydomonas alpha-tubulin is posttranslationally modified by acetylation on the epsilon-amino group of a lysine. Biochemistry.

[41] Bulinski J.C., Gundersen G.G. (1991). Stabilization of post-translational modification of microtubules during cellular morphogenesis. Bioessays.

[42] Hubbert C., Guardiola A., Shao R., Kawaguchi Y., Ito A., Nixon A., Yoshida M., Wang X.F., Yao T.P. (2002). HDAC6 is a microtubule-associated deacetylase. Nature.

[43] North B.J., Marshall B.L., Borra M.T., Denu J.M., Verdin E. (2003). The human Sir2 ortholog, SIRT2, is an NAD+-dependent tubulin deacetylase. Mol. Cell.

[44] Portran D., Schaedel L., Xu Z., Théry M., Nachury M.V. (2017). Tubulin acetylation protects long-lived microtubules against mechanical ageing. Nat. Cell Biol..

[45] Xu Z., Schaedel L., Portran D., Aguilar A., Gaillard J., Marinkovich M.P., Théry M., Nachury M.V. (2017). Microtubules acquire resistance from mechanical breakage through intralumenal acetylation. Science.

[46] Eshun-Wilson L., Zhang R., Portran D., Nachury M.V., Toso D.B., Löhr T., Vendruscolo M., Bonomi M., Fraser J.S., Nogales E. (2019). Effects of α-tubulin acetylation on microtubule structure and stability. Proc. Natl. Acad. Sci. USA.

[47] Even A., Morelli G., Broix L., Scaramuzzino C., Turchetto S., Gladwyn-Ng I., Le Bail R., Shilian M., Freeman S., Magiera M.M. (2019). ATAT1-enriched vesicles promote microtubule acetylation via axonal transport. Sci. Adv..

[48] Bär J., Popp Y., Bucher M., Mikhaylova M. (2022). Direct and indirect effects of tubulin post-translational modifications on microtubule stability: Insights and regulations. Biochim Biophys Acta Mol. Cell Res..

[49] Chu C.W., Hou F., Zhang J., Phu L., Loktev A.V., Kirkpatrick D.S., Jackson P.K., Zhao Y., Zou H. (2011). A novel acetylation of β-tubulin by San modulates microtubule polymerization via down-regulating tubulin incorporation. Mol. Biol. Cell.

[50] Saunders H.A.J., Johnson-Schlitz D.M., Jenkins B.V., Volkert P.J., Yang S.Z., Wildonger J. (2022). Acetylated α-tubulin K394 regulates microtubule stability to shape the growth of axon terminals. Curr. Biol..

[51] Khawaja S., Gundersen G.G., Bulinski J.C. (1988). Enhanced stability of microtubules enriched in detyrosinated tubulin is not a direct function of detyrosination level. J. Cell Biol..

[52] Ersfeld K., Wehland J., Plessmann U., Dodemont H., Gerke V., Weber K. (1993). Characterization of the tubulin-tyrosine ligase. J. Cell Biol..

[53] Aillaud C., Bosc C., Peris L., Bosson A., Heemeryck P., Van Dijk J., Le Friec J., Boulan B., Vossier F., Sanman L.E. (2017). Vasohibins/SVBP are tubulin carboxypeptidases (TCPs) that regulate neuron differentiation. Science.

[54] van der Laan S., Lévêque M.F., Marcellin G., Vezenkov L., Lannay Y., Dubra G., Bompard G., Ovejero S., Urbach S., Burgess A. (2019). Evolutionary Divergence of Enzymatic Mechanisms for Tubulin Detyrosination. Cell Rep..

[55] Rogowski K., van Dijk J., Magiera M.M., Bosc C., Deloulme J.C., Bosson A., Peris L., Gold N.D., Lacroix B., Bosch Grau M. (2010). A family of protein-deglutamylating enzymes associated with neurodegeneration. Cell.

[56] Wu H.Y., Rong Y., Correia K., Min J., Morgan J.I. (2015). Comparison of the enzymatic and functional properties of three cytosolic carboxypeptidase family members. J. Biol. Chem..

[57] Bré M.H., de Néchaud B., Wolff A., Fleury A. (1994). Glutamylated tubulin probed in ciliates with the monoclonal antibody GT335. Cell Motil. Cytoskeleton..

[58] Mary J., Redeker V., Le Caer J.P., Rossier J., Schmitter J.M. (1996). Posttranslational modifications in the C-terminal tail of axonemal tubulin from sea urchin sperm. J. Biol. Chem..

[59] Geimer S., Teltenkötter A., Plessmann U., Weber K., Lechtreck K.F. (1997). Purification and characterization of basal apparatuses from a flagellate green alga. Cell Motil. Cytoskeleton..

[60] Ikegami K., Mukai M., Tsuchida J., Heier R.L., Macgregor G.R., Setou M. (2006). TTLL7 is a mammalian beta-tubulin polyglutamylase required for growth of MAP2-positive neurites. J. Biol. Chem..

[61] Kimura Y., Kurabe N., Ikegami K., Tsutsumi K., Konishi Y., Kaplan O.I., Kunitomo H., Iino Y., Blacque O.E., Setou M. (2010). Identification of tubulin deglutamylase among Caenorhabditis elegans and mammalian cytosolic carboxypeptidases (CCPs). J. Biol. Chem..

[62] Magiera M.M., Janke C. (2013). Investigating tubulin posttranslational modifications with specific antibodies. Methods Cell Biol..

[63] Valenstein M.L., Roll-Mecak A. (2016). Graded Control of Microtubule Severing by Tubulin Glutamylation. Cell.

[64] Kubo T., Oda T. (2019). Chlamydomonas as a tool to study tubulin polyglutamylation. Microscopy.

[65] Lessard D.V., Zinder O.J., Hotta T., Verhey K.J., Ohi R., Berger C.L. (2019). Polyglutamylation of tubulin’s C-terminal tail controls pausing and motility of kinesin-3 family member KIF1A. J. Biol. Chem..

[66] Shin S.C., Im S.K., Jang E.H., Jin K.S., Hur E.M., Kim E.E. (2019). Structural and Molecular Basis for Katanin-Mediated Severing of Glutamylated Microtubules. Cell Rep..

[67] Bodakuntla S., Yuan X., Genova M., Gadadhar S., Leboucher S., Birling M.C., Klein D., Martini R., Janke C., Magiera M.M. (2021). Distinct roles of α- and β-tubulin polyglutamylation in controlling axonal transport and in neurodegeneration. EMBO J..

[68] Song Y., Kirkpatrick L.L., Schilling A.B., Helseth D.L., Chabot N., Keillor J.W., Johnson G.V., Brady S.T. (2013). Transglutaminase and polyamination of tubulin: Posttranslational modification for stabilizing axonal microtubules. Neuron.

[69] Wloga D., Joachimiak E., Fabczak H. (2017). Tubulin Post-Translational Modifications and Microtubule Dynamics. Int. J. Mol. Sci..

[70] Williams S.K., Howarth N.L., Devenny J.J., Bitensky M.W. (1982). Structural and functional consequences of increased tubulin glycosylation in diabetes mellitus. Proc. Natl. Acad. Sci. USA.

[71] McLean W.G. (1997). The role of axonal cytoskeleton in diabetic neuropathy. Neurochem Res..

[72] Fourest-Lieuvin A., Peris L., Gache V., Garcia-Saez I., Juillan-Binard C., Lantez V., Job D. (2006). Microtubule regulation in mitosis: Tubulin phosphorylation by the cyclin-dependent kinase Cdk1. Mol. Biol. Cell..

[73] Ori-McKenney K.M., McKenney R.J., Huang H.H., Li T., Meltzer S., Jan L.Y., Vale R.D., Wiita A.P., Jan Y.N. (2016). Phosphorylation of β-Tubulin by the Down Syndrome Kinase, Minibrain/DYRK1a, Regulates Microtubule Dynamics and Dendrite Morphogenesis. Neuron.

[74] Wandosell F., Serrano L., Avila J. (1987). Phosphorylation of alpha-tubulin carboxyl-terminal tyrosine prevents its incorporation into microtubules. J. Biol. Chem..

[75] Matten W.T., Aubry M., West J., Maness P.F. (1990). Tubulin is phosphorylated at tyrosine by pp60c-src in nerve growth cone membranes. J. Cell Biol..

[76] Koenning M., Wang X., Karki M., Jangid R.K., Kearns S., Tripathi D.N., Cianfrocco M., Verhey K.J., Jung S.Y., Coarfa C. (2021). Neuronal SETD2 activity links microtubule methylation to an anxiety-like phenotype in mice. Brain.

[77] Xie X., Wang S., Li M., Diao L., Pan X., Chen J., Zou W., Zhang X., Feng W., Bao L. (2021). α-TubK40me3 is required for neuronal polarization and migration by promoting microtubule formation. Nat. Commun..

[78] Panse V.G., Hardeland U., Werner T., Kuster B., Hurt E. (2004). A proteome-wide approach identifies sumoylated substrate proteins in yeast. J. Biol. Chem..

[79] Rosas-Acosta G., Russell W.K., Deyrieux A., Russell D.H., Wilson V.G. (2005). A universal strategy for proteomic studies of SUMO and other ubiquitin-like modifiers. Mol. Cell. Proteomics..

[80] Feng W., Liu R., Xie X., Diao L., Gao N., Cheng J., Zhang X., Li Y., Bao L. (2021). SUMOylation of α-tubulin is a novel modification regulating microtubule dynamics. J. Mol. Cell Biol..

[81] Ren Y., Zhao J., Feng J. (2003). Parkin binds to alpha/beta tubulin and increases their ubiquitination and degradation. J. Neurosci..

[82] Wolff J., Zambito A.M., Britto P.J., Knipling L. (2000). Autopalmitoylation of tubulin. Protein Sci..

[83] Wolff J. (2009). Plasma membrane tubulin. Biochim Biophys Acta..

[84] Fukata Y., Dimitrov A., Boncompain G., Vielemeyer O., Perez F., Fukata M. (2013). Local palmitoylation cycles define activity-regulated postsynaptic subdomains. J. Cell Biol..

[85] Rogowski K., Juge F., van Dijk J., Wloga D., Strub J.M., Levilliers N., Thomas D., Bré M.H., Van Dorsselaer A., Gaertig J. (2009). Evolutionary divergence of enzymatic mechanisms for posttranslational polyglycylation. Cell.

[86] Wloga D., Webster D.M., Rogowski K., Bré M.H., Levilliers N., Jerka-Dziadosz M., Janke C., Dougan S.T., Gaertig J. (2009). TTLL3 Is a tubulin glycine ligase that regulates the assembly of cilia. Dev. Cell.

[87] Pathak N., Austin C.A., Drummond I.A. (2011). Tubulin tyrosine ligase-like genes ttll3 and ttll6 maintain zebrafish cilia structure and motility. J. Biol. Chem..

[88] Bosch Grau M., Gonzalez Curto G., Rocha C., Magiera M.M., Marques Sousa P., Giordano T., Spassky N., Janke C. (2013). Tubulin glycylases and glutamylases have distinct functions in stabilization and motility of ependymal cilia. J. Cell Biol..

[89] Barra H.S., Arce C.A., Rodríguez J.A., Caputto R. (1974). Some common properties of the protein that incorporates tyrosine as a single unit and the microtubule proteins. Biochem Biophys Res. Commun..

[90] Nieuwenhuis J., Adamopoulos A., Bleijerveld O.B., Mazouzi A., Stickel E., Celie P., Altelaar M., Knipscheer P., Perrakis A., Blomen V.A. (2017). Vasohibins encode tubulin detyrosinating activity. Science.

[91] Murofushi H. (1980). Purification and characterization of tubulin-tyrosine ligase from porcine brain. J. Biochem..

[92] Magiera M.M., Janke C. (2014). Post-translational modifications of tubulin. Curr. Biol..

[93] Magiera M.M., Singh P., Gadadhar S., Janke C. (2018). Tubulin Posttranslational Modifications and Emerging Links to Human Disease. Cell.

[94] Moutin M.J., Bosc C., Peris L., Andrieux A. (2021). Tubulin post-translational modifications control neuronal development and functions. Dev. Neurobiol..

[95] Kath C., Goni-Oliver P., Müller R., Schultz C., Haucke V., Eickholt B., Schmoranzer J. (2018). PTEN suppresses axon outgrowth by down-regulating the level of detyrosinated microtubules. PLoS ONE.

[96] Gundersen G.G., Kalnoski M.H., Bulinski J.C. (1984). Distinct populations of microtubules: Tyrosinated and nontyrosinated alpha tubulin are distributed differently in vivo. Cell.

[97] Kreitzer G., Liao G., Gundersen G.G. (1999). Detyrosination of tubulin regulates the interaction of intermediate filaments with microtubules in vivo via a kinesin-dependent mechanism. Mol. Biol. Cell.

[98] Peris L., Wagenbach M., Lafanechère L., Brocard J., Moore A.T., Kozielski F., Job D., Wordeman L., Andrieux A. (2009). Motor-dependent microtubule disassembly driven by tubulin tyrosination. J. Cell Biol..

[99] Janke C., Kneussel M. (2010). Tubulin post-translational modifications: Encoding functions on the neuronal microtubule cytoskeleton. Trends Neurosci..

[100] Hammond J.W., Huang C.F., Kaech S., Jacobson C., Banker G., Verhey K.J. (2010). Posttranslational modifications of tubulin and the polarized transport of kinesin-1 in neurons. Mol. Biol. Cell.

[101] Nirschl J.J., Magiera M.M., Lazarus J.E., Janke C., Holzbaur E.L. (2016). α-Tubulin Tyrosination and CLIP-170 Phosphorylation Regulate the Initiation of Dynein-Driven Transport in Neurons. Cell Rep..

[102] Robson S.J., Burgoyne R.D. (1989). Differential localisation of tyrosinated, detyrosinated, and acetylated alpha-tubulins in neurites and growth cones of dorsal root ganglion neurons. Cell Motil. Cytoskeleton..

[103] Brown D.A., Warn R.M. (1993). Primary and secondary chick heart fibroblasts: Fast and slow-moving cells show no significant difference in microtubule dynamics. Cell Motil. Cytoskeleton..

[104] Erck C., Peris L., Andrieux A., Meissirel C., Gruber A.D., Vernet M., Schweitzer A., Saoudi Y., Pointu H., Bosc C. (2005). A vital role of tubulin-tyrosine-ligase for neuronal organization. Proc. Natl. Acad. Sci. USA.

[105] Soppina V., Herbstman J.F., Skiniotis G., Verhey K.J. (2012). Luminal localization of α-tubulin K40 acetylation by cryo-EM analysis of fab-labeled microtubules. PLoS ONE.

[106] Shida T., Cueva J.G., Xu Z., Goodman M.B., Nachury M.V. (2010). The major alpha-tubulin K40 acetyltransferase alphaTAT1 promotes rapid ciliogenesis and efficient mechanosensation. Proc. Natl. Acad. Sci. USA.

[107] Dan W., Gao N., Li L., Zhu J.X., Diao L., Huang J., Han Q.J., Wang S., Xue H., Wang Q. (2018). α-Tubulin Acetylation Restricts Axon Overbranching by Dampening Microtubule Plus-End Dynamics in Neurons. Cereb. Cortex.

[108] Zempel H., Luedtke J., Kumar Y., Biernat J., Dawson H., Mandelkow E., Mandelkow E.M. (2013). Amyloid-β oligomers induce synaptic damage via Tau-dependent microtubule severing by TTLL6 and spastin. EMBO J..

[109] Lacroix B., van Dijk J., Gold N.D., Guizetti J., Aldrian-Herrada G., Rogowski K., Gerlich D.W., Janke C. (2010). Tubulin polyglutamylation stimulates spastin-mediated microtubule severing. J. Cell Biol..

[110] Kuo Y.W., Trottier O., Mahamdeh M., Howard J. (2019). Spastin is a dual-function enzyme that severs microtubules and promotes their regrowth to increase the number and mass of microtubules. Proc. Natl. Acad. Sci. USA.

[111] Park J.H., Roll-Mecak A. (2018). The tubulin code in neuronal polarity. Curr. Opin. Neurobiol..

[112] Audebert S., Koulakoff A., Berwald-Netter Y., Gros F., Denoulet P., Eddé B. (1994). Developmental regulation of polyglutamylated alpha- and beta-tubulin in mouse brain neurons. J. Cell Sci..

[113] Machado A.S., Darmohray D.M., Fayad J., Marques H.G., Carey M.R. (2015). A quantitative framework for whole-body coordination reveals specific deficits in freely walking ataxic mice. Elife.

[114] Shashi V., Magiera M.M., Klein D., Zaki M., Schoch K., Rudnik-Schöneborn S., Norman A., Lopes Abath Neto O., Dusl M., Yuan X. (2018). Loss of tubulin deglutamylase CCP1 causes infantile-onset neurodegeneration. EMBO J..

[115] Kim S.Y., Grant P., Lee J.H., Pant H.C., Steinert P.M. (1999). Differential expression of multiple transglutaminases in human brain. Increased expression and cross-linking by transglutaminases 1 and 2 in Alzheimer’s disease. J. Biol. Chem..

[116] Hadjivassiliou M., Aeschlimann P., Strigun A., Sanders D.S., Woodroofe N., Aeschlimann D. (2008). Autoantibodies in gluten ataxia recognize a novel neuronal transglutaminase. Ann. Neurol..

[117] Encalada S.E., Szpankowski L., Xia C.H., Goldstein L.S. (2011). Stable kinesin and dynein assemblies drive the axonal transport of mammalian prion protein vesicles. Cell.

[118] Sirajuddin M., Rice L.M., Vale R.D. (2014). Regulation of microtubule motors by tubulin isotypes and post-translational modifications. Nat. Cell Biol..

[119] Konishi Y., Setou M. (2009). Tubulin tyrosination navigates the kinesin-1 motor domain to axons. Nat. Neurosci..

[120] Kahn O.I., Sharma V., González-Billault C., Baas P.W. (2015). Effects of kinesin-5 inhibition on dendritic architecture and microtubule organization. Mol. Biol. Cell..

[121] Kubo T., Yanagisawa H.A., Yagi T., Hirono M., Kamiya R. (2010). Tubulin polyglutamylation regulates axonemal motility by modulating activities of inner-arm dyneins. Curr. Biol..

[122] Suryavanshi S., Eddé B., Fox L.A., Guerrero S., Hard R., Hennessey T., Kabi A., Malison D., Pennock D., Sale W.S. (2010). Tubulin glutamylation regulates ciliary motility by altering inner dynein arm activity. Curr. Biol..

[123] McKenney R.J., Huynh W., Vale R.D., Sirajuddin M. (2016). Tyrosination of α-tubulin controls the initiation of processive dynein-dynactin motility. EMBO J..

[124] Ikegami. K., Heier R.L., Taruishi M., Takagi H., Mukai M., Shimma S., Taira S., Hatanaka K., Morone N., Yao I. (2007). Loss of alpha-tubulin polyglutamylation in ROSA22 mice is associated with abnormal targeting of KIF1A and modulated synaptic function. Proc. Natl. Acad. Sci. USA.

[125] Verhey K.J., Gaertig J. (2007). The tubulin code. Cell Cycle.

[126] Tortosa E., Kapitein L.C., Hoogenraad C.C. (2016). Microtubule Organization and Microtubule-Associated Proteins (MAPs). Dendrites.

[127] Kelliher M.T., Saunders H.A., Wildonger J. (2019). Microtubule control of functional architecture in neurons. Curr. Opin. Neurobiol..

[128] Bonnet C., Boucher D., Lazereg S., Pedrotti B., Islam K., Denoulet P., Larcher J.C. (2001). Differential binding regulation of microtubule-associated proteins MAP1A, MAP1B, and MAP2 by tubulin polyglutamylation. J. Biol. Chem..

[129] Morrison E.E. (2007). Action and interactions at microtubule ends. Cell Mol. Life Sci..

[130] Peris L., Thery M., Fauré J., Saoudi Y., Lafanechère L., Chilton J.K., Gordon-Weeks P., Galjart N., Bornens M., Wordeman L. (2006). Tubulin tyrosination is a major factor affecting the recruitment of CAP-Gly proteins at microtubule plus ends. J. Cell Biol..

[131] Bieling P., Kandels-Lewis S., Telley I.A., van Dijk J., Janke C., Surrey T. (2008). CLIP-170 tracks growing microtubule ends by dynamically recognizing composite EB1/tubulin-binding sites. J. Cell Biol..

[132] Badin-Larçon A.C., Boscheron C., Soleilhac J.M., Piel M., Mann C., Denarier E., Fourest-Lieuvin A., Lafanechère L., Bornens M., Job D. (2004). Suppression of nuclear oscillations in Saccharomyces cerevisiae expressing Glu tubulin. Proc. Natl. Acad. Sci. USA.

[133] Nakata T., Hirokawa N. (2003). Microtubules provide directional cues for polarized axonal transport through interaction with kinesin motor head. J. Cell Biol..

[134] Wloga D., Gaertig J. (2010). Post-translational modifications of microtubules. J. Cell Sci..

[135] Lu W., Fox P., Lakonishok M., Davidson M.W., Gelfand V.I. (2013). Initial neurite outgrowth in Drosophila neurons is driven by kinesin-powered microtubule sliding. Curr. Biol..

[136] Tas R.P., Chazeau A., Cloin B.M.C., Lambers M.L.A., Hoogenraad C.C., Kapitein L.C. (2017). Differentiation between Oppositely Oriented Microtubules Controls Polarized Neuronal Transport. Neuron.

[137] Schoenfeld T.A., McKerracher L., Obar R., Vallee R.B. (1989). MAP 1A and MAP 1B are structurally related microtubule associated proteins with distinct developmental patterns in the CNS. J. Neurosci..

[138] Boucher D., Larcher J.C., Gros F., Denoulet P. (1994). Polyglutamylation of tubulin as a progressive regulator of in vitro interactions between the microtubule-associated protein Tau and tubulin. Biochemistry.

[139] Harada A., Teng J., Takei Y., Oguchi K., Hirokawa N. (2002). MAP2 is required for dendrite elongation, PKA anchoring in dendrites, and proper PKA signal transduction. J. Cell Biol..

[140] Brandt R., Lee G. (1993). The balance between tau protein’s microtubule growth and nucleation activities: Implications for the formation of axonal microtubules. J. Neurochem..

[141] Qiang L., Yu W., Andreadis A., Luo M., Baas P.W. (2006). Tau protects microtubules in the axon from severing by katanin. J. Neurosci..

[142] Méphon-Gaspard A., Boca M., Pioche-Durieu C., Desforges B., Burgo A., Hamon L., Piétrement O., Pastré D. (2016). Role of tau in the spatial organization of axonal microtubules: Keeping parallel microtubules evenly distributed despite macromolecular crowding. Cell. Mol. Life Sci..

[143] Hernández-Vega A., Braun M., Scharrel L., Jahnel M., Wegmann S., Hyman B.T., Alberti S., Diez S., Hyman A.A. (2017). Local Nucleation of Microtubule Bundles through Tubulin Concentration into a Condensed Tau Phase. Cell Rep..

[144] Hoogenraad C.C., Akhmanova A., Grosveld F., De Zeeuw C.I., Galjart N. (2000). Functional analysis of CLIP-115 and its binding to microtubules. J. Cell Sci..

[145] Lazarus J.E., Moughamian A.J., Tokito M.K., Holzbaur E.L. (2013). Dynactin subunit p150(Glued) is a neuron-specific anti-catastrophe factor. PLoS Biol..

[146] Errico A., Claudiani P., D’Addio M., Rugarli E.I. (2004). Spastin interacts with the centrosomal protein NA14, and is enriched in the spindle pole, the midbody and the distal axon. Hum. Mol. Genet..

[147] Svenson I.K., Kloos M.T., Jacon A., Gallione C., Horton A.C., Pericak-Vance M.A., Ehlers M.D., Marchuk D.A. (2005). Subcellular localization of spastin: Implications for the pathogenesis of hereditary spastic paraplegia. Neurogenetics.

[148] Sudo H., Baas P.W. (2010). Acetylation of microtubules influences their sensitivity to severing by katanin in neurons and fibroblasts. J. Neurosci..

[149] Gumy L.F., Chew D.J., Tortosa E., Katrukha E.A., Kapitein L.C., Tolkovsky A.M., Hoogenraad C.C., Fawcett J.W. (2013). The kinesin-2 family member KIF3C regulates microtubule dynamics and is required for axon growth and regeneration. J. Neurosci..

[150] Bailey M.E., Sackett D.L., Ross J.L. (2015). Katanin Severing and Binding Microtubules Are Inhibited by Tubulin Carboxy Tails. Biophys. J..

[151] Jaworski J., Kapitein L.C., Gouveia S.M., Dortland B.R., Wulf P.S., Grigoriev I., Camera P., Spangler S.A., Di Stefano P., Demmers J. (2009). Dynamic microtubules regulate dendritic spine morphology and synaptic plasticity. Neuron.

[152] Hu X., Ballo L., Pietila L., Viesselmann C., Ballweg J., Lumbard D., Stevenson M., Merriam E., Dent E.W. (2011). BDNF-induced increase of PSD-95 in dendritic spines requires dynamic microtubule invasions. J. Neurosci..

[153] Yau K.W., van Beuningen S.F., Cunha-Ferreira I., Cloin B.M., van Battum E.Y., Will L., Schätzle P., Tas R.P., van Krugten J., Katrukha E.A. (2014). Microtubule minus-end binding protein CAMSAP2 controls axon specification and dendrite development. Neuron.

[154] Andersen S.S., Bi G.Q. (2000). Axon formation: A molecular model for the generation of neuronal polarity. Bioessays.

[155] Barnes A.P., Polleux F. (2009). Establishment of axon-dendrite polarity in developing neurons. Annu. Rev. Neurosci..

[156] Conde C., Cáceres A. (2009). Microtubule assembly, organization and dynamics in axons and dendrites. Nat. Rev. Neurosci..

[157] Yu W., Baas P.W. (1994). Changes in microtubule number and length during axon differentiation. J. Neurosci..

[158] Roossien D.H., Lamoureux P., Miller K.E. (2014). Cytoplasmic dynein pushes the cytoskeletal meshwork forward during axonal elongation. J. Cell Sci..

[159] Ahmad F.J., Hughey J., Wittmann T., Hyman A., Greaser M., Baas P.W. (2000). Motor proteins regulate force interactions between microtubules and microfilaments in the axon. Nat. Cell Biol..

[160] Ahmad F.J., He Y., Myers K.A., Hasaka T.P., Francis F., Black M.M., Baas P.W. (2006). Effects of dynactin disruption and dynein depletion on axonal microtubules. Traffic.

[161] Myers K.A., Tint I., Nadar C.V., He Y., Black M.M., Baas P.W. (2006). Antagonistic forces generated by cytoplasmic dynein and myosin-II during growth cone turning and axonal retraction. Traffic.

[162] Grabham P.W., Seale G.E., Bennecib M., Goldberg D.J., Vallee R.B. (2007). Cytoplasmic dynein and LIS1 are required for microtubule advance during growth cone remodeling and fast axonal outgrowth. J. Neurosci..

[163] McElmurry K., Stone J.E., Ma D., Lamoureux P., Zhang Y., Steidemann M., Fix L., Huang F., Miller K.E., Suter D.M. (2020). Dynein-mediated microtubule translocation powering neurite outgrowth in chick and Aplysia neurons requires microtubule assembly. J. Cell Sci..

[164] Voelzmann A., Hahn I., Pearce S.P., Sánchez-Soriano N., Prokop A. (2016). A conceptual view at microtubule plus end dynamics in neuronal axons. Brain Res. Bull..

[165] Melkov A., Abdu U. (2018). Regulation of long-distance transport of mitochondria along microtubules. Cell Mol. Life Sci..

[166] Rodríguez-Martín T., Cuchillo-Ibáñez I., Noble W., Nyenya F., Anderton B.H., Hanger D.P. (2013). Tau phosphorylation affects its axonal transport and degradation. NeuroBiol. Aging.

[167] Shahpasand K., Uemura I., Saito T., Asano T., Hata K., Shibata K., Toyoshima Y., Hasegawa M., Hisanaga S. (2012). Regulation of mitochondrial transport and inter-microtubule spacing by tau phosphorylation at the sites hyperphosphorylated in Alzheimer’s disease. J. Neurosci..

[168] Caceres A., Potrebic S., Kosik K.S. (1991). The effect of tau antisense oligonucleotides on neurite formation of cultured cerebellar macroneurons. J. Neurosci..

[169] Sadot E., Barg J., Rasouly D., Lazarovici P., Ginzburg I. (1995). Short- and long-term mechanisms of tau regulation in PC12 cells. J. Cell Sci..

[170] Fukata Y., Itoh T.J., Kimura T., Ménager C., Nishimura T., Shiromizu T., Watanabe H., Inagaki N., Iwamatsu A., Hotani H. (2002). CRMP-2 binds to tubulin heterodimers to promote microtubule assembly. Nat. Cell Biol..

[171] Kimura T., Watanabe H., Iwamatsu A., Kaibuchi K. (2005). Tubulin and CRMP-2 complex is transported via Kinesin-1. J. Neurochem..

[172] Horiguchi K., Hanada T., Fukui Y., Chishti A.H. (2006). Transport of PIP3 by GAKIN, a kinesin-3 family protein, regulates neuronal cell polarity. J. Cell Biol..

[173] Haque S.A., Hasaka T.P., Brooks A.D., Lobanov P.V., Baas P.W. (2004). Monastrol, a prototype anti-cancer drug that inhibits a mitotic kinesin, induces rapid bursts of axonal outgrowth from cultured postmitotic neurons. Cell Motil. Cytoskeleton..

[174] Yoon S.Y., Choi J.E., Huh J.W., Hwang O., Lee H.S., Hong H.N., Kim D. (2005). Monastrol, a selective inhibitor of the mitotic kinesin Eg5, induces a distinctive growth profile of dendrites and axons in primary cortical neuron cultures. Cell Motil. Cytoskeleton..

[175] Bugiel M., Chugh M., Jachowski T.J., Schäffer E., Jannasch A. (2020). The Kinesin-8 Kip3 Depolymerizes Microtubules with a Collective Force-Dependent Mechanism. Biophys J..

[176] Ghosh-Roy A., Goncharov A., Jin Y., Chisholm A.D. (2012). Kinesin-13 and tubulin posttranslational modifications regulate microtubule growth in axon regeneration. Dev. Cell.

[177] Witte H., Neukirchen D., Bradke F. (2008). Microtubule stabilization specifies initial neuronal polarization. J. Cell Biol..

[178] Gonzalez-Billault C., Avila J., Cáceres A. (2001). Evidence for the role of MAP1B in axon formation. Mol. Biol. Cell.

[179] Jacobson C., Schnapp B., Banker G.A. (2006). A change in the selective translocation of the Kinesin-1 motor domain marks the initial specification of the axon. Neuron.

[180] Kahn O.I., Baas P.W. (2016). Microtubules and Growth Cones: Motors Drive the Turn. Trends Neurosci..

[181] Myers K.A., Baas P.W. (2007). Kinesin-5 regulates the growth of the axon by acting as a brake on its microtubule array. J. Cell Biol..

[182] Liu M., Nadar V.C., Kozielski F., Kozlowska M., Yu W., Baas P.W. (2010). Kinesin-12, a mitotic microtubule-associated motor protein, impacts axonal growth, navigation, and branching. J. Neurosci..

[183] Xu M., Liu D., Dong Z., Wang X., Wang X., Liu Y., Baas P.W., Liu M. (2014). Kinesin-12 influences axonal growth during zebrafish neural development. Cytoskelet.

[184] Marcos S., Moreau J., Backer S., Job D., Andrieux A., Bloch-Gallego E. (2009). Tubulin tyrosination is required for the proper organization and pathfinding of the growth cone. PLoS ONE.

[185] Harris W.A., Holt C.E., Bonhoeffer F. (1987). Retinal axons with and without their somata, growing to and arborizing in the tectum of Xenopus embryos: A time-lapse video study of single fibres in vivo. Development.

[186] O’Leary D.D., Bicknese A.R., De Carlos J.A., Heffner C.D., Koester S.E., Kutka L.J., Terashima T. (1990). Target selection by cortical axons: Alternative mechanisms to establish axonal connections in the developing brain. Cold Spring Harb. Symp. Quant. Biol..

[187] Dent E.W., Callaway J.L., Szebenyi G., Baas P.W., Kalil K. (1999). Reorganization and movement of microtubules in axonal growth cones and developing interstitial branches. J. Neurosci..

[188] Dent E.W., Kalil K. (2001). Axon branching requires interactions between dynamic microtubules and actin filaments. J. Neurosci..

[189] Kalil K., Dent E.W. (2014). Branch management: Mechanisms of axon branching in the developing vertebrate CNS. Nat. Rev. Neurosci..

[190] Armijo-Weingart L., Ketschek A., Sainath R., Pacheco A., Smith G.M., Gallo G. (2019). Neurotrophins induce fission of mitochondria along embryonic sensory axons. Elife.

[191] Kalil K., Szebenyi G., Dent E.W. (2000). Common mechanisms underlying growth cone guidance and axon branching. J. Neurobiol..

[192] Yu W., Qiang L., Solowska J.M., Karabay A., Korulu S., Baas P.W. (2008). The microtubule-severing proteins spastin and katanin participate differently in the formation of axonal branches. Mol. Biol. Cell.

[193] Tymanskyj S.R., Yang B., Falnikar A., Lepore A.C., Ma L. (2017). MAP7 Regulates Axon Collateral Branch Development in Dorsal Root Ganglion Neurons. J. Neurosci..

[194] Hooikaas P.J., Martin M., Mühlethaler T., Kuijntjes G.J., Peeters C.A.E., Katrukha E.A., Ferrari L., Stucchi R., Verhagen D.G.F., van Riel W.E. (2019). MAP7 family proteins regulate kinesin-1 recruitment and activation. J. Cell Biol..

[195] Tymanskyj S.R., Ma L. (2019). MAP7 Prevents Axonal Branch Retraction by Creating a Stable Microtubule Boundary to Rescue Polymerization. J. Neurosci..

[196] Goyal U., Renvoisé B., Chang J., Blackstone C. (2014). Spastin-interacting protein NA14/SSNA1 functions in cytokinesis and axon development. PLoS ONE.

[197] Basnet N., Nedozralova H., Crevenna A.H., Bodakuntla S., Schlichthaerle T., Taschner M., Cardone G., Janke C., Jungmann R., Magiera M.M. (2018). Direct induction of microtubule branching by microtubule nucleation factor SSNA1. Nat. Cell Biol..

[198] Baas P.W., Black M.M., Banker G.A. (1989). Changes in microtubule polarity orientation during the development of hippocampal neurons in culture. J. Cell Biol..

[199] Bianchi S., Stimpson C.D., Duka T., Larsen M.D., Janssen W.G., Collins Z., Bauernfeind A.L., Schapiro S.J., Baze W.B., McArthur M.J. (2013). Synaptogenesis and development of pyramidal neuron dendritic morphology in the chimpanzee neocortex resembles humans. Proc. Natl. Acad. Sci. USA.

[200] Baas P.W., Lin S. (2011). Hooks and comets: The story of microtubule polarity orientation in the neuron. Dev. Neurobiol..

[201] Kollins K.M., Bell R.L., Butts M., Withers G.S. (2009). Dendrites differ from axons in patterns of microtubule stability and polymerization during development. Neural Dev..

[202] Hill S.E., Parmar M., Gheres K.W., Guignet M.A., Huang Y., Jackson F.R., Rolls M.M. (2012). Development of dendrite polarity in Drosophila neurons. Neural Dev..

[203] Yau K.W., Schätzle P., Tortosa E., Pagès S., Holtmaat A., Kapitein L.C., Hoogenraad C.C. (2016). Dendrites In Vitro and In Vivo Contain Microtubules of Opposite Polarity and Axon Formation Correlates with Uniform Plus-End-Out Microtubule Orientation. J. Neurosci..

[204] Sharp D.J., Kuriyama R., Essner R., Baas P.W. (1997). Expression of a minus-end-directed motor protein induces Sf9 cells to form axon-like processes with uniform microtubule polarity orientation. J. Cell Sci..

[205] Yu W., Cook C., Sauter C., Kuriyama R., Kaplan P.L., Baas P.W. (2000). Depletion of a microtubule-associated motor protein induces the loss of dendritic identity. J. Neurosci..

[206] Yu W., Lu B. (2012). Synapses and dendritic spines as pathogenic targets in Alzheimer’s disease. Neural Plast..

[207] Sánchez-Huertas C., Freixo F., Viais R., Lacasa C., Soriano E., Lüders J. (2016). Non-centrosomal nucleation mediated by augmin organizes microtubules in post-mitotic neurons and controls axonal microtubule polarity. Nat. Commun..

[208] Cunha-Ferreira I., Chazeau A., Buijs R.R., Stucchi R., Will L., Pan X., Adolfs Y., van der Meer C., Wolthuis J.C., Kahn O.I. (2018). The HAUS Complex Is a Key Regulator of Non-centrosomal Microtubule Organization during Neuronal Development. Cell Rep..

[209] Liang X., Kokes M., Fetter R.D., Sallee M.D., Moore A.W., Feldman J.L., Shen K. (2020). Growth cone-localized microtubule organizing center establishes microtubule orientation in dendrites. Elife..

[210] Yoong L.F., Lim H.K., Tran H., Lackner S., Zheng Z., Hong P., Moore A.W. (2020). Atypical Myosin Tunes Dendrite Arbor Subdivision. Neuron.

[211] Vaillant A.R., Zanassi P., Walsh G.S., Aumont A., Alonso A., Miller F.D. (2002). Signaling mechanisms underlying reversible, activity-dependent dendrite formation. Neuron.

[212] Getz S.A., Tariq K., Marchand D.H., Dickson C.R., Howe V.J.R., Skelton P.D., Wang W., Li M., Barry J.M., Hong J. (2022). PTEN Regulates Dendritic Arborization by Decreasing Microtubule Polymerization Rate. J. Neurosci..

[213] Delandre C., Amikura R., Moore A.W. (2016). Microtubule nucleation and organization in dendrites. Cell Cycle.

[214] Gu J., Firestein B.L., Zheng J.Q. (2008). Microtubules in dendritic spine development. J. Neurosci..

[215] Kapitein L.C., Yau K.W., Hoogenraad C.C. (2010). Microtubule dynamics in dendritic spines. Methods Cell Biol..

[216] van Rossum D., Kuhse J., Betz H. (1999). Dynamic interaction between soluble tubulin and C-terminal domains of N-methyl-D-aspartate receptor subunits. J. Neurochem..

[217] Sheng M., Hoogenraad C.C. (2007). The postsynaptic architecture of excitatory synapses: A more quantitative view. Annu. Rev. Biochem..

[218] Chicurel M.E., Terrian D.M., Potter H. (1993). mRNA at the synapse: Analysis of a synaptosomal preparation enriched in hippocampal dendritic spines. J. Neurosci..

[219] Gu J., Zheng J.Q. (2009). Microtubules in Dendritic Spine Development and Plasticity. Open NeuroSci. J..

[220] Hu X., Viesselmann C., Nam S., Merriam E., Dent E.W. (2008). Activity-dependent dynamic microtubule invasion of dendritic spines. J. Neurosci..

[221] Schätzle P., Esteves da Silva M., Tas R.P., Katrukha E.A., Hu H.Y., Wierenga C.J., Kapitein L.C., Hoogenraad C.C. (2018). Activity-Dependent Actin Remodeling at the Base of Dendritic Spines Promotes Microtubule Entry. Curr. Biol..

[222] Zhang W., Cheng L.E., Kittelmann M., Li J., Petkovic M., Cheng T., Jin P., Guo Z., Göpfert M.C., Jan L.Y. (2015). Ankyrin Repeats Convey Force to Gate the NOMPC Mechanotransduction Channel. Cell.

[223] Yan C., Wang F., Peng Y., Williams C.R., Jenkins B., Wildonger J., Kim H.J., Perr J.B., Vaughan J.C., Kern M.E. (2018). Microtubule Acetylation Is Required for Mechanosensation in Drosophila. Cell Rep..

[224] McVicker D.P., Awe A.M., Richters K.E., Wilson R.L., Cowdrey D.A., Hu X., Chapman E.R., Dent E.W. (2016). Transport of a kinesin-cargo pair along microtubules into dendritic spines undergoing synaptic plasticity. Nat. Commun..

[225] Brandt R., Lee G. (1994). Orientation, assembly, and stability of microtubule bundles induced by a fragment of tau protein. Cell Motil. Cytoskeleton..

[226] Guzik B.W., Goldstein L.S. (2004). Microtubule-dependent transport in neurons: Steps towards an understanding of regulation, function and dysfunction. Curr. Opin Cell Biol..

[227] Yogev S., Cooper R., Fetter R., Horowitz M., Shen K. (2016). Microtubule Organization Determines Axonal Transport Dynamics. Neuron.

[228] Buscaglia G., Northington K.R., Moore J.K., Bates E.A. (2020). Reduced TUBA1A Tubulin Causes Defects in Trafficking and Impaired Adult Motor Behavior. eNeuro.

[229] Bora G., Hensel N., Rademacher S., Koyunoğlu D., Sunguroğlu M., Aksu-Mengeş E., Balcı-Hayta B., Claus P., Erdem-Yurter H. (2021). Microtubule-associated protein 1B dysregulates microtubule dynamics and neuronal mitochondrial transport in spinal muscular atrophy. Hum. Mol. Genet..

[230] Bodakuntla S., Schnitzler A., Villablanca C., Gonzalez-Billault C., Bieche I., Janke C., Magiera M.M. (2020). Tubulin polyglutamylation is a general traffic-control mechanism in hippocampal neurons. J. Cell Sci..

[231] Gilmore-Hall S., Kuo J., Ward J.M., Zahra R., Morrison R.S., Perkins G., La Spada A.R. (2019). CCP1 promotes mitochondrial fusion and motility to prevent Purkinje cell neuron loss in pcd mice. J. Cell Biol..

[232] Reed N.A., Cai D., Blasius T.L., Jih G.T., Meyhofer E., Gaertig J., Verhey K.J. (2006). Microtubule acetylation promotes kinesin-1 binding and transport. Curr. Biol..

[233] Monroy B.Y., Tan T.C., Oclaman J.M., Han J.S., Simó S., Niwa S., Nowakowski D.W., McKenney R.J., Ori-McKenney K.M. (2020). A Combinatorial MAP Code Dictates Polarized Microtubule Transport. Dev. Cell.

[234] Monroy B.Y., Sawyer D.L., Ackermann B.E., Borden M.M., Tan T.C., Ori-McKenney K.M. (2018). Competition between microtubule-associated proteins directs motor transport. Nat. Commun..

[235] Sferra A., Fattori F., Rizza T., Flex E., Bellacchio E., Bruselles A., Petrini S., Cecchetti S., Teson M., Restaldi F. (2018). Defective kinesin binding of TUBB2A causes progressive spastic ataxia syndrome resembling sacsinopathy. Hum. Mol. Genet..

[236] Uchimura S., Oguchi Y., Katsuki M., Usui T., Osada H., Nikawa J., Ishiwata S., Muto E. (2006). Identification of a strong binding site for kinesin on the microtubule using mutant analysis of tubulin. EMBO J..

[237] Niwa S., Takahashi H., Hirokawa N. (2013). β-Tubulin mutations that cause severe neuropathies disrupt axonal transport. EMBO J..

[238] Silva M., Morsci N., Nguyen K.C.Q., Rizvi A., Rongo C., Hall D.H., Barr M.M. (2017). Cell-Specific α-Tubulin Isotype Regulates Ciliary Microtubule Ultrastructure, Intraflagellar Transport, and Extracellular Vesicle Biology. Curr. Biol..

[239] Leterrier C., Vacher H., Fache M.P., d’Ortoli S.A., Castets F., Autillo-Touati A., Dargent B. (2011). End-binding proteins EB3 and EB1 link microtubules to ankyrin G in the axon initial segment. Proc. Natl. Acad. Sci. USA.

[240] Hatten M.E. (2002). New directions in neuronal migration. Science.

[241] Kapitein L.C., Hoogenraad C.C. (2015). Building the Neuronal Microtubule Cytoskeleton. Neuron.

[242] Fry A.E., Cushion T.D., Pilz D.T. (2014). The genetics of lissencephaly. Am. J. Med Genet C Semin Med Genet..

[243] Bizzotto S., Francis F. (2015). Morphological and functional aspects of progenitors perturbed in cortical malformations. Front. Cell Neurosci..

[244] Solecki D.J., Model L., Gaetz J., Kapoor T.M., Hatten M.E. (2004). Par6alpha signaling controls glial-guided neuronal migration. Nat. Neurosci..

[245] Umeshima H., Hirano T., Kengaku M. (2007). Microtubule-based nuclear movement occurs independently of centrosome positioning in migrating neurons. Proc. Natl. Acad. Sci. USA.

[246] Tsai J.W., Bremner K.H., Vallee R.B. (2007). Dual subcellular roles for LIS1 and dynein in radial neuronal migration in live brain tissue. Nat. Neurosci..

[247] Tanaka T., Serneo F.F., Higgins C., Gambello M.J., Wynshaw-Boris A., Gleeson J.G. (2004). Lis1 and doublecortin function with dynein to mediate coupling of the nucleus to the centrosome in neuronal migration. J. Cell Biol..

[248] Wu Y.K., Kengaku M. (2018). Dynamic Interaction between Microtubules and the Nucleus Regulates Nuclear Movement During Neuronal Migration. J. Exp. Neurosci..

[249] Ori-McKenney K.M., Vallee R.B. (2011). Neuronal migration defects in the Loa dynein mutant mouse. Neural Dev..

[250] Hirotsune S., Fleck M.W., Gambello M.J., Bix G.J., Chen A., Clark G.D., Ledbetter D.H., McBain C.J., Wynshaw-Boris A. (1998). Graded reduction of Pafah1b1 (Lis1) activity results in neuronal migration defects and early embryonic lethality. Nat. Genet..

[251] Gleeson J.G., Lin P.T., Flanagan L.A., Walsh C.A. (1999). Doublecortin is a microtubule-associated protein and is expressed widely by migrating neurons. Neuron.

[252] Moores C.A., Perderiset M., Kappeler C., Kain S., Drummond D., Perkins S.J., Chelly J., Cross R., Houdusse A., Francis F. (2006). Distinct roles of doublecortin modulating the microtubule cytoskeleton. EMBO J..

[253] Couillard-Despres S., Winner B., Schaubeck S., Aigner R., Vroemen M., Weidner N., Bogdahn U., Winkler J., Kuhn H.G., Aigner L. (2005). Doublecortin expression levels in adult brain reflect neurogenesis. Eur. J. Neurosci..

[254] Kappeler C., Saillour Y., Baudoin J.P., Tuy F.P., Alvarez C., Houbron C., Gaspar P., Hamard G., Chelly J., Métin C. (2006). Branching and nucleokinesis defects in migrating interneurons derived from doublecortin knockout mice. Hum. Mol. Genet..

[255] Di Donato N., Timms A.E., Aldinger K.A., Mirzaa G.M., Bennett J.T., Collins S., Olds C., Mei D., Chiari S., Carvill G. (2018). Analysis of 17 genes detects mutations in 81% of 811 patients with lissencephaly. Genet. Med..

[256] Subramanian L., Calcagnotto M.E., Paredes M.F. (2020). Cortical Malformations: Lessons in Human Brain Development. Front. Cell. Neurosci..

[257] Bahi-Buisson N., Poirier K., Boddaert N., Saillour Y., Castelnau L., Philip N., Buyse G., Villard L., Joriot S., Marret S. (2008). Refinement of cortical dysgeneses spectrum associated with TUBA1A mutations. J. Med Genet..

[258] Poirier K., Lebrun N., Broix L., Tian G., Saillour Y., Boscheron C., Parrini E., Valence S., Pierre B.S., Oger M. (2013). Mutations in TUBG1, DYNC1H1, KIF5C and KIF2A cause malformations of cortical development and microcephaly. Nat. Genet..

[259] Bahi-Buisson N., Poirier K., Fourniol F., Saillour Y., Valence S., Lebrun N., Hully M., Bianco C.F., Boddaert N., Elie C. (2014). The wide spectrum of tubulinopathies: What are the key features for the diagnosis?. Brain.

[260] Fallet-Bianco C., Laquerrière A., Poirier K., Razavi F., Guimiot F., Dias P., Loeuillet L., Lascelles K., Beldjord C., Carion N. (2014). Mutations in tubulin genes are frequent causes of various foetal malformations of cortical development including microlissencephaly. Acta Neuropathol. Commun..

[261] Aiken J., Moore J.K., Bates E.A. (2019). TUBA1A mutations identified in lissencephaly patients dominantly disrupt neuronal migration and impair dynein activity. Hum. Mol. Genet..

[262] Myers K.A., Bello-Espinosa L.E., Kherani A., Wei X.C., Innes A.M. (2015). TUBA1A Mutation Associated with Eye Abnormalities in Addition to Brain Malformation. Pediatr. Neurol..

[263] Yokoi S., Ishihara N., Miya F., Tsutsumi M., Yanagihara I., Fujita N., Yamamoto H., Kato M., Okamoto N., Tsunoda T. (2015). TUBA1A mutation can cause a hydranencephaly-like severe form of cortical dysgenesis. Sci. Rep..

[264] Hebebrand M., Hüffmeier U., Trollmann R., Hehr U., Uebe S., Ekici A.B., Kraus C., Krumbiegel M., Reis A., Thiel C.T. (2019). The mutational and phenotypic spectrum of TUBA1A-associated tubulinopathy. Orphanet J. Rare Dis..

[265] Löwe J., Li H., Downing K.H., Nogales E. (2001). Refined structure of alpha beta-tubulin at 3.5 A resolution. J. Mol. Biol..

[266] Leca I., Phillips A.W., Hofer I., Landler L., Ushakova L., Cushion T.D., Dürnberger G., Stejskal K., Mechtler K., Keays D.A. (2020). A proteomic survey of microtubule-associated proteins in a R402H TUBA1A mutant mouse. PLoS Genet..

[267] Keays D.A., Tian G., Poirier K., Huang G.J., Siebold C., Cleak J., Oliver P.L., Fray M., Harvey R.J., Molnár Z. (2007). Mutations in alpha-tubulin cause abnormal neuronal migration in mice and lissencephaly in humans. Cell.

[268] Belvindrah R., Natarajan K., Shabajee P., Bruel-Jungerman E., Bernard J., Goutierre M., Moutkine I., Jaglin X.H., Savariradjane M., Irinopoulou T. (2017). Mutation of the α-tubulin Tuba1a leads to straighter microtubules and perturbs neuronal migration. J. Cell Biol..

[269] Jansen A., Andermann E. (2005). Genetics of the polymicrogyria syndromes. J. Med Genet..

[270] Jaglin X.H., Poirier K., Saillour Y., Buhler E., Tian G., Bahi-Buisson N., Fallet-Bianco C., Phan-Dinh-Tuy F., Kong X.P., Bomont P. (2009). Mutations in the beta-tubulin gene TUBB2B result in asymmetrical polymicrogyria. Nat. Genet..

[271] Cederquist G.Y., Luchniak A., Tischfield M.A., Peeva M., Song Y., Menezes M.P., Chan W.M., Andrews C., Chew S., Jamieson R.V. (2012). An inherited TUBB2B mutation alters a kinesin-binding site and causes polymicrogyria, CFEOM and axon dysinnervation. Hum. Mol. Genet..

[272] Guerrini R., Mei D., Cordelli D.M., Pucatti D., Franzoni E., Parrini E. (2012). Symmetric polymicrogyria and pachygyria associated with TUBB2B gene mutations. Eur. J. Hum. Genet..

[273] Poirier K., Saillour Y., Bahi-Buisson N., Jaglin X.H., Fallet-Bianco C., Nabbout R., Castelnau-Ptakhine L., Roubertie A., Attie-Bitach T., Desguerre I. (2010). Mutations in the neuronal ß-tubulin subunit TUBB3 result in malformation of cortical development and neuronal migration defects. Hum. Mol. Genet..

[274] Moffat J.J., Ka M., Jung E.M., Kim W.Y. (2015). Genes and brain malformations associated with abnormal neuron positioning. Mol. Brain.

[275] Barkovich A.J., Kuzniecky R.I., Jackson G.D., Guerrini R., Dobyns W.B. (2001). Classification system for malformations of cortical development: Update 2001. Neurology.

[276] Verrotti A., Spalice A., Ursitti F., Papetti L., Mariani R., Castronovo A., Mastrangelo M., Iannetti P. (2010). New trends in neuronal migration disorders. Eur. J. Paediatr. Neurol..

